# Organochloride mediated prodrug activation induced by ionizing radiation

**DOI:** 10.1039/d5sc03191e

**Published:** 2025-11-24

**Authors:** Juncheng Liu, Bing Xu, Mark A. R. de Geus, Antonia G. Denkova, Rienk Eelkema

**Affiliations:** a Department of Chemical Engineering, Delft University of Technology van der Maasweg 9 2629 HZ Delft Netherlands R.Eelkema@tudelft.nl; b Department of Radiation Science and Technology, Delft University of Technology Mekelweg 15 2629 JB Delft Netherlands A.G.Denkova@tudelft.nl

## Abstract

Boronic acid and ester-caged prodrugs have been widely investigated in cellular-generated hydrogen peroxide triggered release. Although it is well-known that ionizing radiation generates hydrogen peroxide in aqueous solution, using this approach to activate boronic acid or ester-based prodrugs suffers from low H_2_O_2_ yields and thus low uncaging efficiency. However, the organochloride peroxyl radical formed from irradiating an aqueous solution of an organochloride may increase the uncaging efficiency. In this study, we used a boronic acid-caged coumarin derivative to quantify the yield of oxidation induced by clinical doses of radiation (less than 8 Gy), and boronic acid-caged gemcitabine to assess the activation of a prodrug upon irradiation. Irradiation of the coumarin derivative in phosphate buffered saline shows a low yield of 0.048 µM per Gy, and the prodrug after irradiation has only limited toxicity to the U87 cell line, indicating limited uncaging. The oxidation of boronic acid can be greatly enhanced by the peroxyl radical generated from irradiation of dilute PBS-organochloride solutions, with the yield increasing to 0.13 µM per Gy. Moreover, the oxidation by peroxyl radical can be catalyzed by *N*,*N*-dimethylaniline derivatives, increasing the yield to 0.19 µM per Gy. Clinical dose irradiation of the caged gemcitabine derivative in a solution of PBS with trichloroethanol and 2-(dimethylamino)benzoic acid shows efficient tumor cell killing and a comparable toxicity with that of the parent drug, indicating efficient uncaging.

## Abstract 2

Radiation-generated organochloride-peroxyl radicals trigger the uncaging of boronic acid prodrugs. Amine catalysis further enhances yields and enables efficient *in vitro* activation of a gemcitabine prodrug at clinical radiation doses.

## Graphical abstract

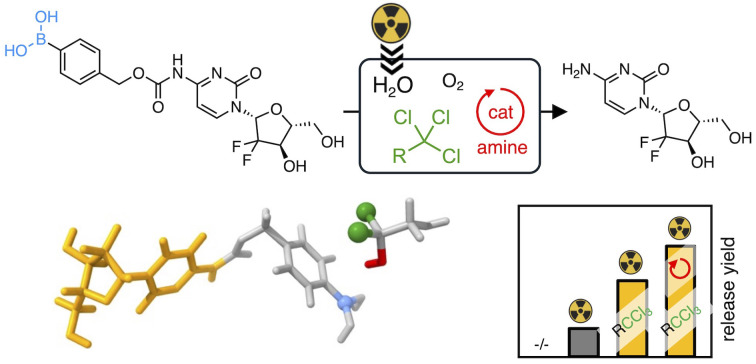

## Introduction

Radiotherapy and chemotherapy are two common approaches to treat malignant tumors. High-energy X-rays or γ-rays are applied in radiotherapy to damage the DNA of cancer cells, while cytotoxic drugs are applied in chemotherapy. Combinations of radiotherapy and chemotherapy have shown enhanced treatment effects for a variety of cancer types, but this strategy is still limited by the systemic toxicity of most chemotherapeutics.^1^ Targeted prodrug activation is a strategy to reduce systemic toxicity. Prodrugs are chemically modified drugs where a key functional group is caged by a cleavable group. These prodrugs are activated by specific stimuli present in the tumor microenvironment (*e.g.* oxidative stress^2,3^ and enzymes^4^) or by external stimuli such as near-infrared light.^5^ Upon activation, the drug is released at the targeted site, enabling precise control over drug delivery. Ionizing radiation-activated prodrugs have been intensively studied in recent years for potential application in combined radiotherapy and chemotherapy with reduced side effects.^6–9^ Since most radiation initiated chemical reactions are mediated by species from water radiolysis,^10^ radiation-sensitive prodrugs should be designed to react with these species (*e.g.* hydrogen radical,^11^ hydroxyl radical^12^ and aqueous electrons^13–17^). However, these species have low radiolytic yield (0.28 µM per Gy for aqueous electron and hydroxyl radical) and the total administered dose in radiation therapy is typically less than 60 Gy, given in 1.8–2 Gy fractions.^18^ In addition, the reactive species react unselectively with cellular components, which often results in a low efficiency of drug activation.

**Fig. 1:**
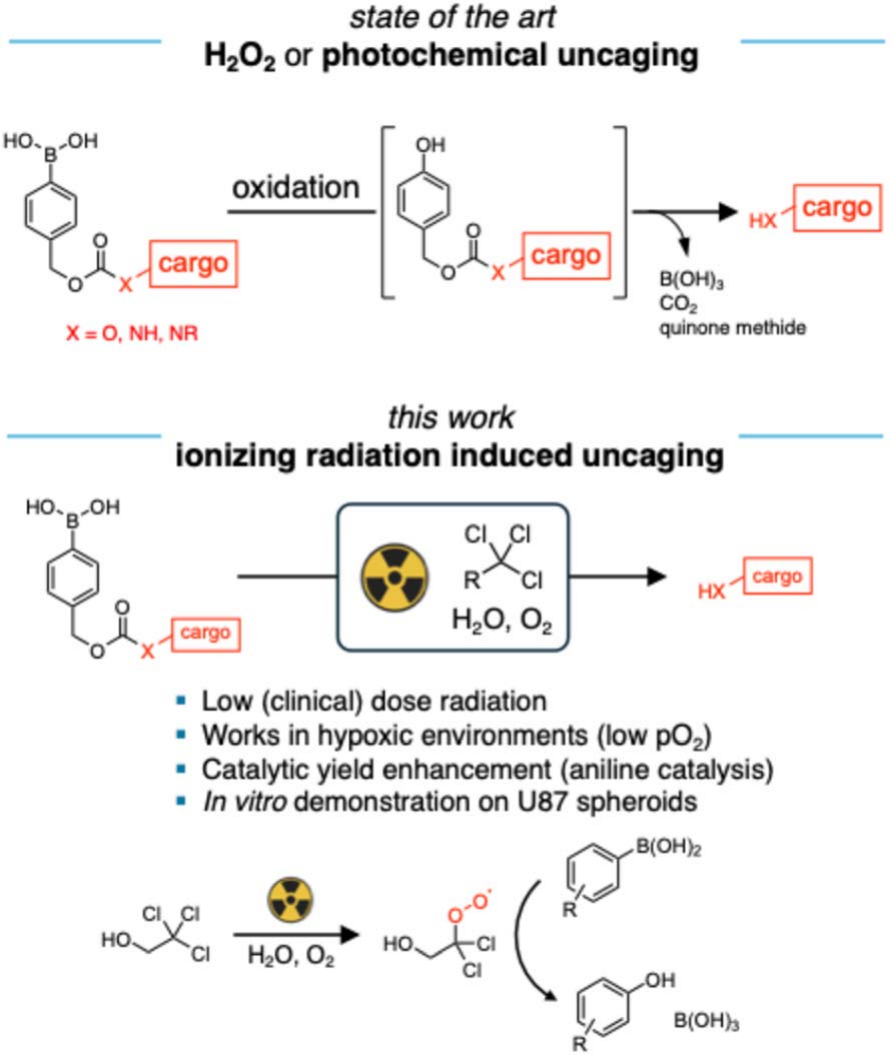
Oxidation of benzylboronic acid cages induces release of hydroxyl or amine cargo. State of the art: oxidation induced by H_2_O_2_ or photochemistry; this work: oxidation induced by ionizing radiation mediated by organochloride additives.

Arylboronic acids or esters are widely used as caging groups in prodrug design and chemical sensing systems, owing to their selective oxidation by reactive oxygen species (ROS) such as hydrogen peroxide (H_2_O_2_) and peroxynitrite,^19,20^ leading to phenol formation through hydrolysis of the intermediate (Fig. 1). Since cancerous cells have high levels of H_2_O_2_ (ranging from 10 µM to 100 µM) compared to normal cells, using intracellularly generated H_2_O_2_ to activate boronic acid-based prodrugs has been reported in anti-cancer therapy.^3,21,22^ Matsushita *et al.* reported a prodrug where the 4-amino group of gemcitabine was caged with an arylboronate-ester-based carbamate group.^2^ Upon oxidation by H_2_O_2_, the boronate hydrolyses, leaving a phenol group that undergoes quinone-methide elimination to release a molecule of CO_2_ and free gemcitabine. However, not all types of cancer overproduce H_2_O_2_, for instance, pancreatic epithelial cell line and human glioblastoma cell line (U87) are not responsive to boronate ester or acid-based prodrugs.^2,3^ Besides, the sluggish reaction rate of H_2_O_2_ and arylboronates lowers the efficiency of prodrug activation.^20,23^ Ionizing radiation, delivered during radiotherapy, generates H_2_O_2_ within tissues, offering potential for the development of radiation-activated prodrugs. However, the practical application of this approach is limited by the generated low yield of H_2_O_2_. Recent years have shown the emergence of alternative strategies for *in vivo* activation of arylboronic acid based prodrugs, in particular through photochemistry using Ir or Os based photocatalysts.^24–26^

In previous work, we demonstrated the oxidation of stilbene and thioether derivatives through irradiation in aerated water containing organochloride.^27,28^ Aqueous electrons generated from water radiolysis can react with the organochloride to form a carbon-centered radical and a chloride ion. The radical reacts rapidly with dissolved oxygen to form a peroxyl radical, a strong oxidant which can be used for subsequent reactions. Here, we present the oxidation of arylboronic acids using ionizing radiation. A fluorescent probe, 7-amino-4-methylcoumarin (AMC), was employed to quantify this reaction since the oxidation of arylboronic acid led to the activation of fluorescence emission. We found that the peroxyl radical generated from the radiolysis of water in the presence of organochloride can efficiently oxidize arylboronic acids (Fig. 1). Furthermore, this oxidation process can be catalyzed by *N*,*N*-dimethylaniline derivatives. Building on these findings, we synthesized a gemcitabine prodrug in which the gemcitabine 4-amino group is protected as an arylboronic acid-based carbamate. Upon exposure to 6 Gy X-rays in phosphate buffer saline (PBS) containing organochloride and the *N*,*N*-dimethylaniline derivative, the prodrug demonstrated an inhibitory effect on U87 spheroid growth. Our findings indicate that the activation of a boronic acid-caged prodrug *via* radiation in the presence of organochlorides is an effective approach for combining chemotherapy with radiation therapy.

## Results and discussion

**Fig. 2:**
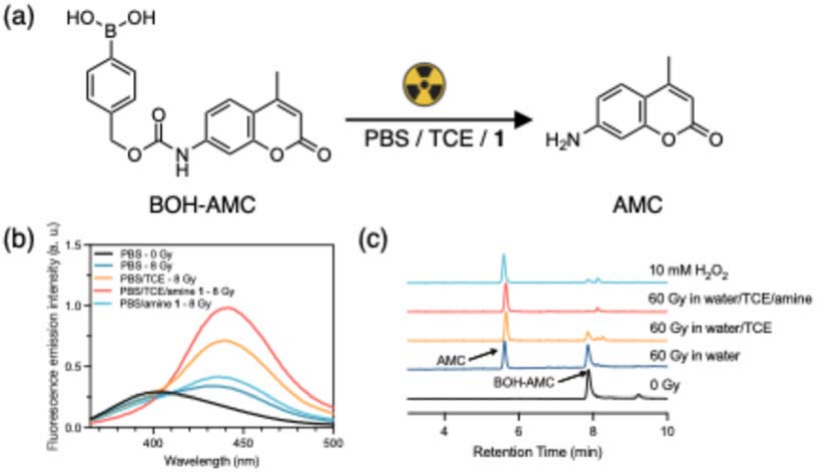
(a) Radiation induced uncaging of BOH-AMC, yielding the AMC fluorescent probe. (b) Fluorescence emission spectrum of BOH-AMC before and after 8 Gy of γ-irradiation (excitation at 330 nm; 10 µM probe in PBS, pH 7.4; 12 mM of TCE if present; 100 µM of amine 1 if present; samples were incubated for 30 min after irradiation); (c) HPLC-UV chromatograph determines the oxidation of BOH-AMC (10 µM in water; 12 mM of TCE if present; 100 µM of amine 1 if present; samples were incubated for 30 min after irradiation).

**Fig. 3:**
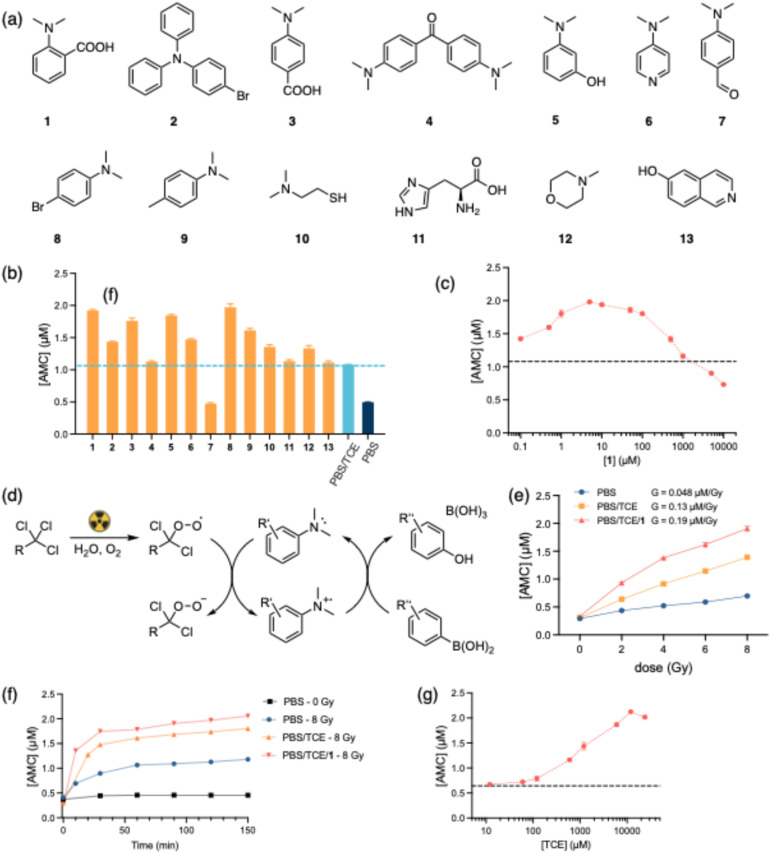
(a) Structure screen of tertiary amines; (b) the concentration of released AMC after exposure to 8 Gy γ-radiation (1–13, irradiation in PBS/12 mM TCE/100 µM amines, dashed line presents the AMC release after 8 Gy in PBS/12 mM TCE); (c) determination of AMC release against [1] (radiation dose, 8 Gy; 10 µM BOH-AMC in PBS/12 mM TCE; dashed line represents the released AMC without 1; *n* = 3 independent experiments); (d) proposed mechanism of *N*,*N*-dimethylaniline catalyzed arylboronic acid oxidation; (e) dose-dependent AMC release induced by radiation (*n* = 3 independent experiments; for some data, error bars are smaller than data markers); (f) time evolution of the probe solutions post-irradiation (radiation dose, 8 Gy; 10 µM probe in PBS, pH 7.4; 12 mM TCE if present; 100 µM of 1 if present); (g) determination of AMC release against [TCE] (radiation dose, 8 Gy; 10 µM probe in PBS/5 µM 1; dashed line represents the released AMC without TCE; *n* = 3 independent experiments).

To study the oxidation of arylboronic acids, we synthesized a 7-amino-4-methylcoumarin based fluorescent probe, in which the 7-amino group is protected by a benzylboronic acid based self-immolative caging group (denoted as BOH-AMC, Fig. 2). The fluorescence emission of AMC is quenched when the 7-amino is caged by an electron-withdrawing carbamate group. Oxidation of boronic acid will lead to the release of the amino group of AMC, coupled to an increase in fluorescence emission. As shown in Fig. 2a, irradiation (8 Gy) of BOH-AMC in PBS leads to an increase in emission intensity at 441 nm, indicating the release of AMC. The yield of AMC is 0.048 µM per Gy in PBS (Fig. 3e), which is close to the G-value (radiolytic yield) of H_2_O_2_ (0.073 µM per Gy). We therefore attribute this release to the oxidation of arylboronic acid by radiation-generated H_2_O_2_. The presence of 2,2,2-trichloroethanol (TCE) during irradiation results in substantially more AMC activation (G-value = 0.13 µM per Gy) as evidenced by a higher emission intensity than that in PBS alone (Fig. 2a and 3e). The key role of TCE implies that the oxidation is caused by the organochloride peroxyradical species generated from irradiating PBS/TCE.^27,28^

To further increase the uncaging efficiency, we looked at alternative means to oxidize boronic acids. It is reported that organic *N*-oxides can oxidize boronic acids or esters selectively and efficiently.^29–33^ Additionally, *N*-oxide derivatives are commonly synthesized by oxidizing tertiary amines using peroxides such as hydrogen peroxide and peroxybenzoic acids.^34^ Building on this, we were curious if tertiary amines can act as mediators of short lived radiation-generated ROS in the uncaging of benzylboronic acids, thereby enhancing the radiolytic uncaging yield. As shown in Fig. 2b, more AMC is activated when 2-(dimethylamino)benzoic acid 1 is present in PBS/TCE during irradiation. In contrast, irradiation of BOH-AMC in PBS/1 results in much lower AMC activation than observed in PBS/TCE/1, indicating that the presence of TCE is crucial in the oxidation process and that radiation-generated ˙OH plays only a minor role, if any. This conclusion is corroborated by high-performance liquid chromatography (HPLC) measurements (Fig. 2c), where irradiation in water/TCE/1 results in the highest conversion from BOH-AMC to AMC among all the irradiated groups.

We investigated a series of tertiary amine categories, including derivatives of *N*,*N*-dimethylaniline (compound 1, 3–9), aliphatic amines (compound 10, 12) and aromatic heterocyclic amines (compound 11, 13) (Fig. 3a) with solutions of BOH-AMC (10 µM) in PBS/12 mM TCE. After irradiation with 8 Gy of γ-radiation, the solutions were incubated at 37 °C for 30 minutes. The emission intensities of AMC are converted to concentrations according to a calibration curve as shown in Fig. S1. In all cases, amine concentrations were 100 µM. As shown in Fig. 3b, irradiation in the presence of 1, 2, 3, 5, 6, 8, 9, 10, 12 leads to enhanced AMC release, whereas the presence of 7 exhibits inhibitory effects. *N*,*N*-dimethylaniline derivatives (1, 3, 5, 8, 9) show more pronounced enhancement compared to aliphatic amines (10, 12) whereas heterocyclic amines (11, 13) show no enhancement.

To test whether *N*,*N*-dimethylanilines serve as a catalyst in the oxidation process, BOH-AMC solutions were prepared in PBS/TCE with varying concentrations of amine 1. The release of AMC was monitored following irradiation (8 Gy). As shown in Fig. 3c, the AMC release exhibits an increase with increasing [1], peaking at 5 µM, after which it decreases. At [1] exceeding ∼1 mM, the activation of AMC decreases even below that observed without the presence of 1. Notably, at [1] = 0.1 µM, approximately 0.36 µM more AMC is activated compared to that in the absence of the amine, suggesting multiple catalytic turnovers by the amine. Our observations align with findings reported by Zhu *et al.*,^32^ wherein *N*,*N*-dimethylaniline *N*-oxide derivatives exhibit the highest reactivity towards arylboronic acids, while aliphatic tertiary amines, such as *N*-methylmorpholine *N*-oxide and trimethylamine *N*-oxide, exhibit sluggish reactivity. In addition, we also detected the *N*-oxide of 9 after irradiation of a water/TCE/9 solution (120 Gy, Fig. S5). However, when we mixed the separately synthesized *N*-oxide of 9 with BOH-AMC and allowed it to react, we did not observe activation of AMC, suggesting a different catalytic mechanism (Fig. S7 and S8). Experiments where we irradiated PBS solutions of TCE or TCE/9 and only after irradiation added the BOH-AMC probe showed much lower levels of uncaging than when the probe was present in the mixture during irradiation (Fig. S9–S11). This result indicates that highly reactive and unstable species are involved in boronic acid oxidation, and that the relatively stable *N*-oxide does not play a significant role. *N*,*N*-dimethylanilines are easily oxidized to their radical cations. These unstable species likely play a role in the catalytic boronic acid uncaging, possibly through cleavage of the carbon-boron^35^ bond and subsequent phenol formation through reaction of the aryl radical^24^ with O_2_. We thereby proposed a catalytic cycle (Fig. 3d) in which peroxyradicals, generated during the irradiation of PBS/TCE, oxidize *N,N*-dimethylaniline to its corresponding radical cation, which then oxidizes the arylboronic acid.

We used compound 1 for subsequent studies. Fig. 3e shows the release AMC profiles in relation to absorbed radiation dose. BOH-AMC (10 µM) was irradiated in PBS, PBS/TCE and PBS/TCE/1, and the solutions were incubated for 30 minutes at room temperature. Irradiation in PBS gives the lowest G-value (0.048 µM per Gy). The presence of 12 mM TCE increases the G-value of AMC to 0.13 µM per Gy, and the G-value is further enhanced (0.19 µM per Gy) when amine 1 is present (Fig. 3e). When measuring the concentration of activated AMC after irradiation, we observed concentration-time profiles that correlate with the G-values associated with the various conditions (Fig. 3f). This observation indicates that, in the two-step process of boronic acid oxidation and subsequent elimination of the 4-hydroxybenzyl carbamate intermediate, the latter step is rate limiting, obscuring measurements of the relative rates of H_2_O_2_ and peroxyradical boronic acid oxidations.^36^ In addition, in the absence of irradiation, BOH-AMC remains stable over the course of the experiment.

**Fig. 4:**
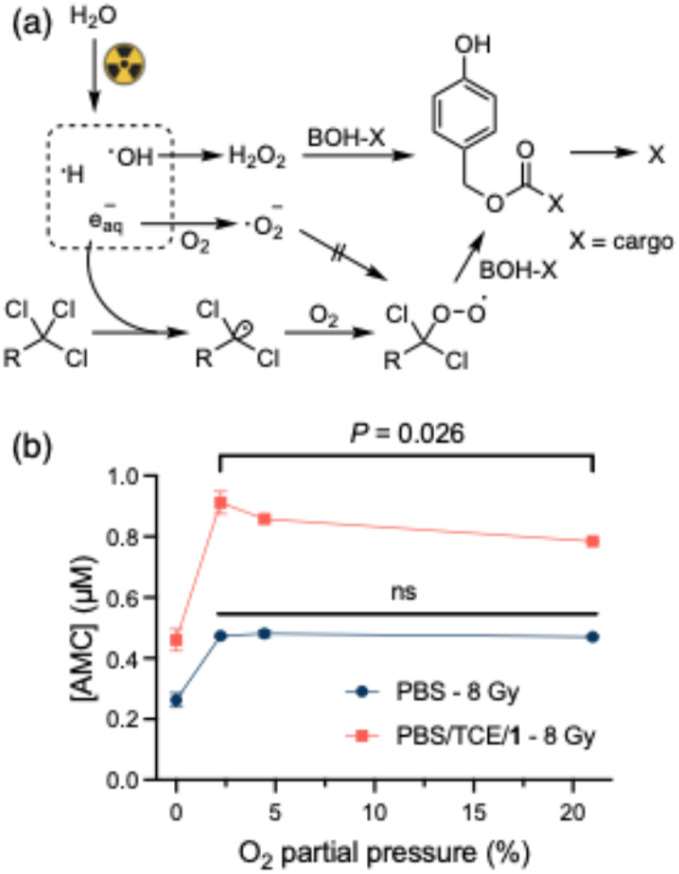
(a) Proposed reaction network, including the competitive effect of oxygen on the boronic acid oxidation; (b) the effect of oxygen concentration on AMC release (*t*-test, *n* = 3, *P* > 0.05 show as “ns”).

The use of organochlorides like TCE in biomedical settings raises concerns about potential hepatotoxicity and carcinogenicity.^37^ It is advisable to minimize TCE usage to mitigate any side effects. Considering this, we investigated the oxidation of BOH-AMC in PBS/TCE/1 with TCE concentrations ranging from 12 µM to 24 mM. As illustrated in Fig. 3g, after exposure to the same dose of γ-irradiation, low concentrations of TCE lead to decreasing release of AMC, while at 24 mM TCE, the release of AMC is slightly lower than that at 12 mM. The strong dependence on TCE concentration can be attributed to the short lifetime of the aqueous electron, where higher TCE concentrations favor the TCE-aqueous electron reaction over reacting with molecular oxygen or other scavengers (Fig. 4a).

It is interesting that molecular oxygen, a crucial species in the formation of the peroxyl radical, can also efficiently scavenge aqueous electrons. To describe the competitive relationship between aqueous electrons, organochloride and molecular oxygen, we proposed a reaction network as depicted in Fig. 4a. There, the organochloride reacts with an aqueous electron to form a carbon-centered radical and a chloride anion. The reaction rate is unknown but is estimated to be diffusion controlled (*k* ∼10^10^ M^−1^ s^−1^).^38^ The formed carbon centered radical reacts with molecular oxygen to form the peroxyl radical (*k* ∼10^9^ M^−1^ s^−1^).^39^ In parallel, H_2_O_2_ is formed primarily by recombination of hydroxyl radicals (*k* = 5.5 × 10^9^ M^−1^ s^−1^),^40^ but the radiolytic yield is considerably lower than that of hydrated electrons and even of the TCE-mediated BOH-AMC uncaging (G(H_2_O_2_) = 0.073 µM per Gy, G(e_aq_^−^) = 0.28 µM per Gy, G(TCE) = 0.13 µM per Gy). Ultimately, either H_2_O_2_ or the organochloride peroxyradical will oxidize the boronic acid, leading to uncaging. They will do so at different rates, depending on their respective concentrations and rate constants, with the H_2_O_2_-mediated oxidation typically proceeding at *k* ∼2 M^−1^ s^−1^ at physiological pH.^41^

Aqueous electrons can react with molecular oxygen to form superoxide anion (*k* = 1.9 × 10^10^ M^−1^ s^−1^)^38^ which is known to be unreactive toward organochlorides.^42^ The comparable reaction rate of oxygen and organochloride with aqueous electrons makes the yield of the peroxyl radical strongly dependent on the concentration ratio of organochloride and oxygen.^43^ To experimentally investigate this dependence and to test if radiation-induced oxidation can be performed under hypoxic conditions, BOH-AMC solutions (10 µM) were irradiated with 8 Gy γ-rays in varying oxygen concentrations. The concentrations of TCE and amine 1 were 120 µM and 5 µM, respectively. Oxygen partial pressure at 2% is the commonly reported value for hypoxic tumors and 5% oxygen is the average for healthy tissues.^44,45^ As shown in Fig. 4b, in the group of irradiation in the presence of TCE and amine 1, the maximum AMC release is observed at 2% (*ca.* 27 µM) oxygen, and higher oxygen concentration results in reduced release of AMC. This result is in line with the proposed mechanism, and means that the oxidation is most efficient under hypoxic conditions. Notably, the release of AMC in the group without organochloride and amine present has a lower total yield which reaches a plateau from 2% oxygen onwards.

**Fig. 5:**
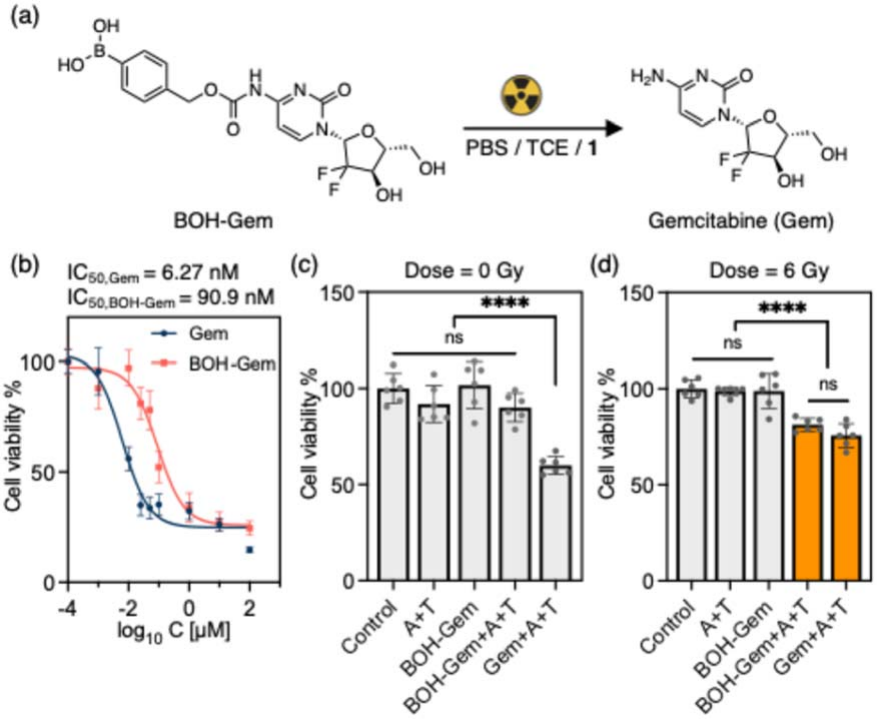
(a) Radiation induced uncaging of benzylboronic acid protected prodrug BOH-Gem to release gemcitabine (Gem); (b) IC_50_ assays of Gem and BOH-Gem on U87 cell line; (c) cell viability of U87 cells without irradiation; (d) cell viability of U87 cells after 6 Gy of X-ray irradiation (control: addition of DMEM; A + T: addition of 5 µM amine 1 and 500 µM TCE; BOH-Gem: addition of 20 nM prodrug; BOH-Gem + A + T: addition of 20 nM prodrug, 5 µM amine 1 and 500 µM TCE; Gem + A + T: addition of 20 nM gemcitabine, 5 µM amine 1 and 500 µM TCE. One-way ANOVA, *n* = 6, *P*-values > 0.05 show as “ns”, ****P < 0.0001).

**Fig. 6:**
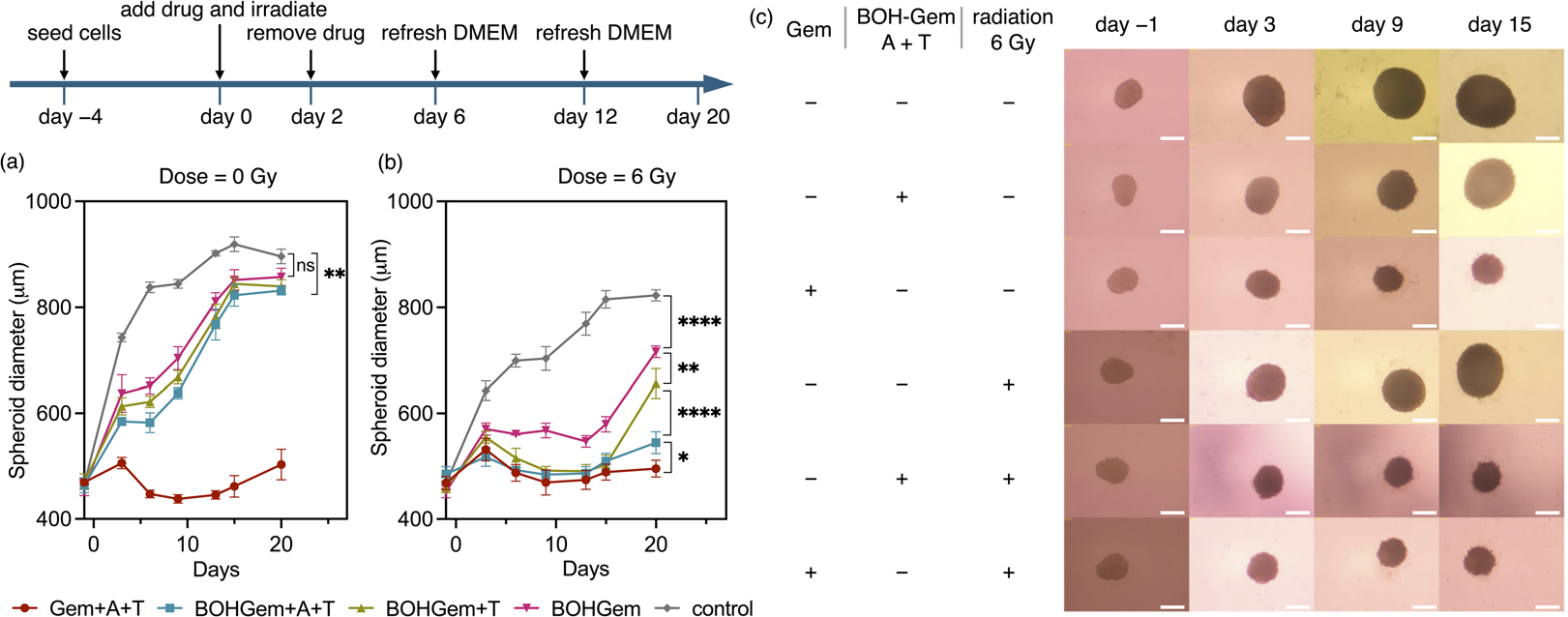
The diameter of spheroids after various treatments as a function of time, shown for samples that are (a) not irradiated and (b) irradiated with 6 Gy X-rays; Gem + A + T: addition of 81 nM gemcitabine, 5 µM amine 1 and 500 µM TCE; BOH-Gem + A + T: addition of 81 nM prodrug, 5 µM amine 1 and 500 µM TCE; BOH-Gem + T: addition of 81 nM prodrug and 500 µM TCE; BOH-Gem: addition of 81 nM prodrug; control: addition of 5 µM amine 1 and 500 µM TCE. One-way ANOVA, *n* = 4, *P < 0.05, **P < 0.01, ****P < 0.0001. (c) Light microscopy images of spheroids treated with indicated conditions, scale bar = 400 µm. (Gem represents addition of 81 nM gemcitabine; BOH-Gem A + T represents addition of 81 nM BOH-Gem, 5 µM amine 1 and 500 µM TCE).

Radiation-initiated activation of prodrugs in a cell environment was tested using gemcitabine (Gem) as the drug. The combination of Gem and radiotherapy has been investigated to treat a variety of cancers since Gem is a potential radiosensitizer *in vitro* and *in vivo*.^46,47^ However, the side effects are unacceptable when Gem is used in combination with radiotherapy or other chemotherapeutics.^48^ Inspired by Matsushita *et al.*,^2^ we synthesized a prodrug, BOH-Gem, where the 4-amino group of Gem was caged by a benzylboronic acid-based carbamate group (Fig. 5a). U87, a human glioblastoma cell line, was selected because of the low level of in-cell generated H_2_O_2_.^3^ We found that the half maximal inhibitory concentration (IC_50_) of BOH-Gem is 90.9 nM against the U87 cell line, which is much higher than that of Gem (6.27 nM) (Fig. 6b). We selected the concentration of 20 nM for both Gem and BOH-Gem in the irradiation experiment. It should be noted that TCE is the active intermediate of chloral hydrate when this is used as sedative agent.^49^ In the following cell experiments, we use TCE at 500 µM concentration, as a proof of concept study. As this concentration is well above the human toxicity limit of TCE, future clinical translation will require other sources of organochloride or covalent attachment to polymeric scaffolds.^28^ Amine 1 is reported to have anti-cancer activity at concentrations above 50-100 µM, without affecting healthy cells.^50^ We used X-rays (240 kV) instead of γ-rays for the radiation source, as the X-rays set-up allowed more control over the precise radiation dose given. Although the radiolytic yield of aqueous electrons is slightly different compared to using γ-rays, the mechanism of prodrug activation will be the same.^51^ The activation of BOH-Gem under various conditions, characterized using the HPLC, is shown in Fig. S2–S4. Exposure to X-rays (60 Gy) in PBS results in partial conversion of BOH-Gem (50 µM) to Gem (Fig. S3). The addition of TCE significantly enhances the conversion, and in the presence of both amine 1 and TCE, BOH-Gem undergoes nearly complete conversion. 6 Gy irradiation of a 10 µM BOH-Gem solution shows the same trend, albeit at an overall lower conversion due to the lower radiation dose (Fig. S4).

As shown in Fig. 5c and d, 5 µM of amine 1 and 500 µM TCE is not toxic toward U87 cells with or without exposure to irradiation, as seen by the non-significant changes in cell viability compared to the control group. Irradiation of cells with BOH-Gem alone does not lead to a high cell killing efficiency (Fig. 5d), which indicates the H_2_O_2_ generated by 6 Gy of X-ray is not enough to release significant amounts of Gem and kill the cells. Irradiation of BOH-Gem with addition of compound 1 and TCE results in a similar cell killing efficiency compared to irradiation of Gem, demonstrating the radiation-driven activation of prodrug causes a comparable toxicity as the parent drug. U87 cells treated with BOH-AMC, TCE, amine 1 and 20 Gy of X-ray radiation demonstrated higher fluorescence intensity compared to that without radiation (Fig. S12), further validating the radiation-induced release of caged cargo.

Having found reduced viability of 2D cell cultures upon prodrug activation, we continued testing with 3D tumor spheroids, which resemble much better the physiological environment of tumors. Tumor spheroids naturally have gradient decrease of oxygen concentration from the periphery to the core.^52–54^ For example, for spheroids of *ca.* 400 nm diameter under standard culture conditions (20% oxygen in gas phase), the oxygen concentration decreases from *ca.* 100 µM on the periphery to nearly zero at the center of spheroids.^53^ Using spheroids as *in vitro* 3D tumor model has provided key insights for understanding hypoxia-related resistance to radiotherapy and chemotherapy.^55,56^ Although a hypoxic microenvironment makes cells resistant to ionizing radiation, it could facilitate organochloride mediated oxidation, thereby enhancing radiation-triggered drug activation (see above).

U87 cells were seeded and allowed to grow for 4 days at which point the diameter was *ca.* 400 µm. Gemcitabine or BOH-Gem (with amine 1 and TCE) was administered on day 0, and radiation was applied on the same day. After treatment on day 0, the spheroids were incubated for 2 days and the cell culture medium (DMEM) was refreshed to remove the drug. The spheroid growth curves and the taken images are shown in Fig. 6. Without drug or radiation, the spheroid diameter increased linearly with time until day 6, after which the growth slowed down and reached a plateau (883 ± 22 µm on day 20). The surface continued to be smooth up to the last day of measurement, day 15 (Fig. 6c). Gemcitabine (81 nM) inhibited spheroid growth (Fig. 6a), with a noticeably smaller diameter than the control on day 3 and shrinkage from day 3 to day 5, after which the growth remained minimal. The surface of the spheroids appeared to be fuzzy on day 15, which indicates decreased intercellular interaction because of dead cells. A lower concentration of gemcitabine (54 nM, Fig. S13a) resulted in an initial growth curve similar to the 81 nM group, followed by apparent regrowth from day 13 to day 20. High concentration of gemcitabine (122 nM, Fig. S13c) caused no growth over the observed period. BOH-Gem is much less effective on growth inhibition than gemcitabine. The growth of spheroids treated with BOH-Gem (81 nM) was slowed from day 3 to day 6, but recovered to linear growth from day 6 and reached plateau from day 15 on, showing no significant difference compared to control on day 20 (Fig. 6a). The starting time of spheroid regrowth was delayed at higher concentration of BOH-Gem, from day 3 at 54 nM to day 9 at 122 nM BOH-Gem (Fig. S13a and c). The addition of 5 µM amine 1 and 500 µM TCE together with BOH-Gem had a slightly inhibition on the growth of spheroids compared to control on day 20 (Fig. 6a).

X-ray irradiation (6 Gy) slightly inhibited spheroid growth. Under these conditions, the spheroids grew to 813 ± 18 µm by day 20, and their surface was smooth on day 15 (Fig. 6c). Irradiating (6 Gy) spheroids that were treated with 81 nM BOH-Gem showed delayed growth where the diameter did not increase from day 3 to day 13 and increased linearly with time from day 13 to day 20 (Fig. 6b). The presence of 500 µM TCE enhanced the inhibiting effect, as evidenced by smaller diameter than the BOH-Gem treated group. The spheroid growth was significantly inhibited by day 20 when spheroids treated with 81 nM BOH-Gem, 5 µM amine 1 and 500 µM TCE were irradiated with 6 Gy of X-rays. The growth curve was similar to the group treated with X-rays and gemcitabine. The spheroids treated with X-rays, BOH-Gem, TCE and amine 1 also developed a fuzzy surface by day 15, which is similar to the group treated with X-rays and gemcitabine (Fig. 6c). The enhancing effect of TCE and amine 1 was more significant at 54 nM BOH-Gem, while irradiating 122 nM BOH-Gem resulted in comparable toxicity as gemcitabine (122 nM) treated group.

## Conclusions

We show that an arylboronic acid-based probe and prodrug can be oxidized by peroxyl radicals generated from the radiolysis of aqueous solutions containing an organochloride compound. The oxidation is substantially enhanced when *N*,*N*-dimethylaniline catalysts are present. Higher concentration of organochloride results in more oxidation, while at low concentrations of organochloride, the yield of oxidizing product depends on the concentration of oxygen. A boronic acid-caged gemcitabine prodrug shows reduced toxicity compared to parent drug. Exposure of prodrug solutions containing an organochloride and *N*,*N*-dimethylaniline derivative to clinical doses of radiation results in comparable cell toxicity as the parent drug and results in growth inhibition of U87 spheroid tumor models. This study demonstrates that the widely applied aryl boronic acid caging group can be removed using ionizing radiation as a trigger, and that this uncaging strategy is effective in cellular environments, using clinical doses of radiation.

## Author contributions

J. L.: conceptualization; investigation; formal analysis; writing – original draft; B. X.: investigation; formal analysis; M. A. R. d. G.: investigation; formal analysis; A. G. D.: supervision; writing – review & editing; R. E.: conceptualization; formal analysis; writing – original draft; writing – review & editing.

## Conflicts of interest

The authors declare no conflicts of interest.

## Supplementary Material

SC-017-D5SC03191E-s001

## Data Availability

Data supporting this study are available from the 4TU. Researchdata repository at https://doi.org/10.4121/6244da9d-d869-4596-b922-a35ce39ff123.v1. Supplementary information (SI): experimental details on the synthesis of BOH-AMC and BOH-Gem; LC-MS and mass spectra; method of oxygen concentration determination; confocal laser scanning microscope images. See DOI: https://doi.org/10.1039/d5sc03191e.
