# Supplementary material for: Organochloride mediated prodrug activation induced by ionizing radiation
Source: Chem Sci. 2025 Nov 24;17(3):1752–60. doi: 10.1039/d5sc03191e (PMC12667067; doi:10.1039/d5sc03191e)
Supplement: SC-017-D5SC03191E-s001 [file SC-017-D5SC03191E-s001.pdf]

Supplementary Information (SI) for Chemical Science.
This journal is © The Royal Society of Chemistry 2025

**Organochloride mediated prodrug activation induced by ionizing radiation**

Juncheng Liu[1,2], Bing Xu[1,2], Mark A. R. de Geus[1], Antonia G. Denkova[2,*] and Rienk Eelkema[1,*]

[1] Department of Chemical Engineering, Delft University of Technology, van der Maasweg 9, 2629 HZ Delft, The Netherlands

[2] Department of Radiation Science and Technology, Delft University of Technology, Mekelweg 15, 2629 JB Delft, The Netherlands

*Corresponding Authors: Antonia G. Denkova and Rienk Eelkema, Email: A.G.Denkova@tudelft.nl; R.Eelkema@tudelft.nl

## Methods

All solvents and chemicals were purchased from commercial suppliers (Sigma Aldrich, Tokyo Chemical Industry and abcr Gute Chemie) and used without further purification. Reactions were monitored by thin-layer chromatography (TLC) on a silica gel plate and visualized by UV light (254 nm). Flash column chromatography was carried out on a 30 cm column loaded with 230-400 mesh silica gel. $^{1}$H-NMR and $^{13}$C-NMR were recorded on an Agilent-400 MR DD2 at 298 K. Fluorescence spectra were recorded with Spex Fluorolog-3 equipped with a standard 90° setup.

**Irradiation methods**: The irradiations with $\gamma$-rays were performed using a Nordion 220 $^{60}$Co gamma cell. The dose rate at the experimental date was around 0.108 Gy/s which is calculated based on the decay law and the half-life of $^{60}$Co. The delivered dose was calculated by the dose rate at the date of the experiments multiplied by the exposure time. The X-ray irradiation was carried out using an X-ray source (Philips MCN 321 variable-energy X-ray tube) performed in a working voltage of 240 keV and a current of 10 mA. The dose rate of the X-ray source was determined using the method as described in literature.[1]

**HPLC-MS Method 1**: HPLC-MS was performed on a LTQ XL electrospray ionization source-mass spectrometer that was connected with a Shimadzu setup with a D2 detector and Discovery C18 reverse phase column. Water/MeCN with 0.1% (v/v) formic acid was used as the mobile phase at a flow rate of 0.2 mL/min.

**HPLC-MS Method 2**: HPLC-UV/MS traces were recorded on a Shimadzu instrument consisting of the following modules: CBM-40 (system controller), LC-20AT (prominence liquid chromatograph), CTO-40C (column oven), SIL-40C (autosampler), LC-40D x 2 (solvent delivery module), SPD-M40 (photodiode array detector), FCV-20AH$_2$ (valve unit) and LCMS-2020 (electrospray ionization mass spec).

Analytical samples were collected at a rate of 15.0 µL/s into a 20 µL sample loop and loaded onto an analytical column (Agilent Zorbax 300SB-C18, 5 µM, 4.6 x 250 mm). Analysis occurred with a flow rate of 1.0 mL/min using the following gradient: A: 0.1% TFA in $H_2O$; B: 0.1% TFA in MeCN. Gradient program: 00.0 – 04.0 min 5% B; 04.0 – 14.5 min 5% to 95% B (linear gradient); 14.5 – 16.0 min 95% B; 16.0 – 17.0 min 95% B to 5% B (linear gradient); 17.0 – 20.0 min 5% B (equilibrate). UV chromatograms were recorded between 0.00 and 15.0 min. MS spectra (ESI in positive mode) were recorded between 04.0 and 15.0 min, with a delay of approximately 30 seconds between the recording of the UV chromatograms and the MS spectra.

**Synthesis of BOH-AMC**

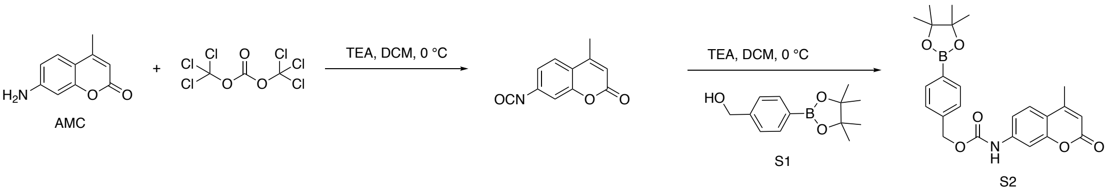


To a flame dried flask was added AMC (1 g, 5.71 mmol, 1.0 eq.), triphosgene (559 mg, 1.88 mmol, 0.3 eq.) and 20 mL DCM. The mixture was cooled to 0 °C using ice bath followed by dropwise adding triethylamine (TEA, 2.37 mL, 17.12 mmol, 3.0 eq.). Compound S1 (1.5 g, 6.28 mmol, 1.1 eq.) was added to the reacting mixture and stirred for overnight at room temperature. The solvent was evaporated under reduced pressure. The crude product was purified by flash chromatography over a silica column, eluted with PE: EA from 20:1 to 1:1, to yield a white powder (1.45 g, 58.4%). $^{1}H$ NMR (400 MHz, $CDCl_3$) δ 7.83 (d, *J* = 8.1 Hz, 2H), 7.52 (d, *J* = 8.6 Hz, 1H), 7.44 – 7.36 (m, 4H), 6.97 (s, 1H), 6.19 (d, *J* = 1.3 Hz, 1H), 5.24 (s, 2H), 2.40 (d, *J* = 1.2 Hz, 3H), 1.34 (s, 13H).
$^{13}C$ NMR (101 MHz, $CDCl_3$) δ 161.16, 154.61, 152.82, 152.29, 141.40, 138.64, 135.27, 127.57, 125.52, 115.76, 114.51, 113.40, 106.11, 84.07, 67.50, 29.85, 25.00, 18.72. MS (ESI+) *m/z* $[M + H]^+$ calculated for $[C_{24}H_{27}BNO_6]^+$, 436.19, found 436.10.

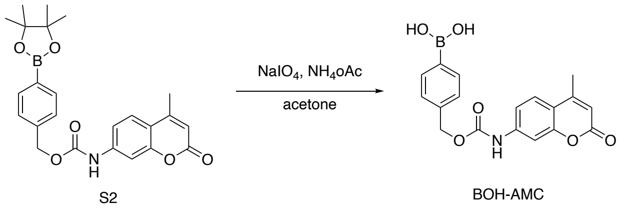


Compound S2 (70.0 mg, 0.16 mmol, 1 eq.) was added to 5 mL acetone followed by adding $NaIO_4$, (172.0 mg, 0.80 mmol, 5 eq.) and ammonium acetate (61.7 mg, 0.80 mmol, 5 eq.) in 2 mL deionized water. The mixture was stirred for 16 hours followed by removal of acetone under reduced pressure. The white precipitate was collected and washed with deionized water 3 times, to yield a white powder (35.0 mg, 61.9%). $^{1}H$ NMR (400 MHz, MeOD) δ 7.80 – 7.56 (m, 4H), 7.48 – 7.32 (m, 3H), 6.20 (s, 1H), 5.23 (s, 2H), 2.45 (s, 3H).
$^{13}C$ NMR (101 MHz, MeOD) δ 163.43, 155.63, 155.43, 155.21, 144.40, 139.22, 135.14, 134.77, 128.18, 128.03, 126.74 (x2), 116.21, 115.90, 112.97, 106.30, 67.73, 18.51. MS (ESI+) *m/z* $[M + H]^+$ calculated for $[C_{18}H_{17}BNO_6]^+$, 354.11, found 354.12.

HPLC, UV (220 nm) (HPLC Method 2). The peak at 3.1 min is associated to the injection with a cosolvent.

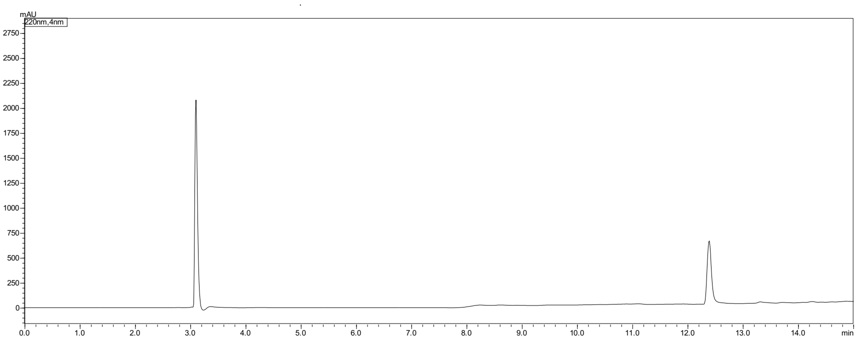

HPLC, UV (330 nm) (HPLC Method 2)

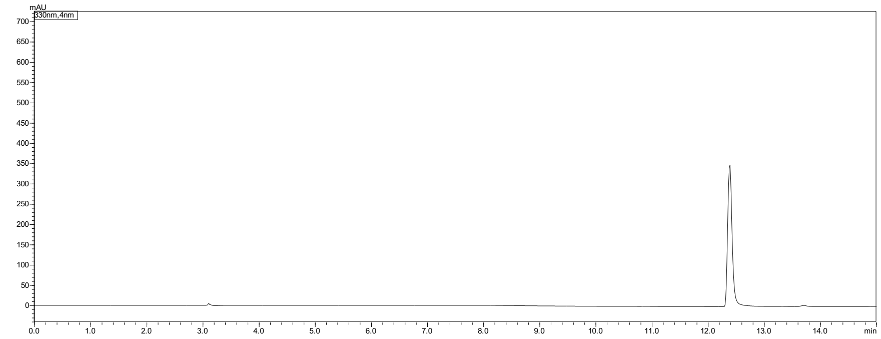

MS, Total Ion Count (TIC) (HPLC Method 2)

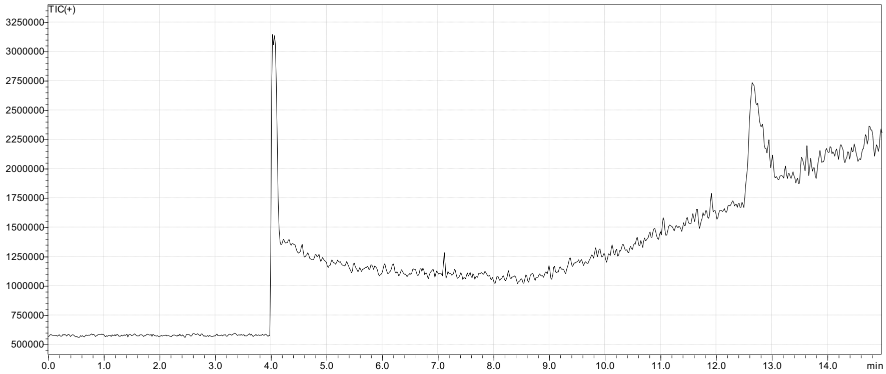

(ESI+) *m/z* $[M + H]^+$ calculated for $[C_{18}H_{17}BNO_6]^+$, 354.1, found 354.1.

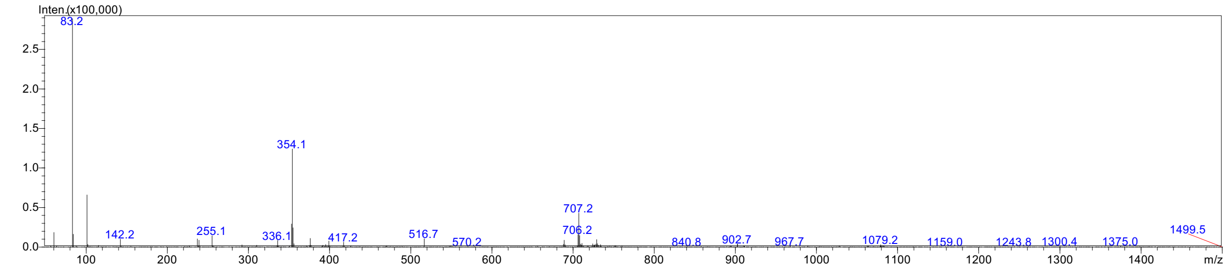

$^{1}$H-NMR (400MHz, D4-MeOD) spectrum of BOH-AMC

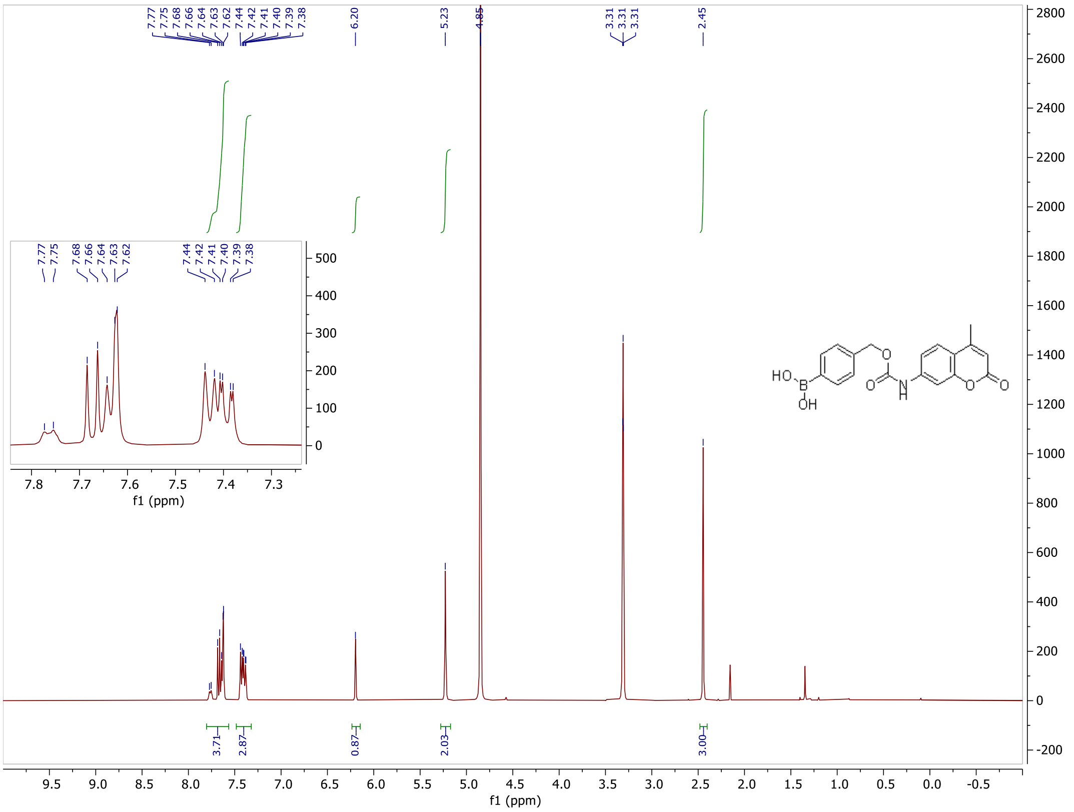


$^{13}$C-NMR (101 MHz, D4-MeOD) spectrum of BOH-AMC

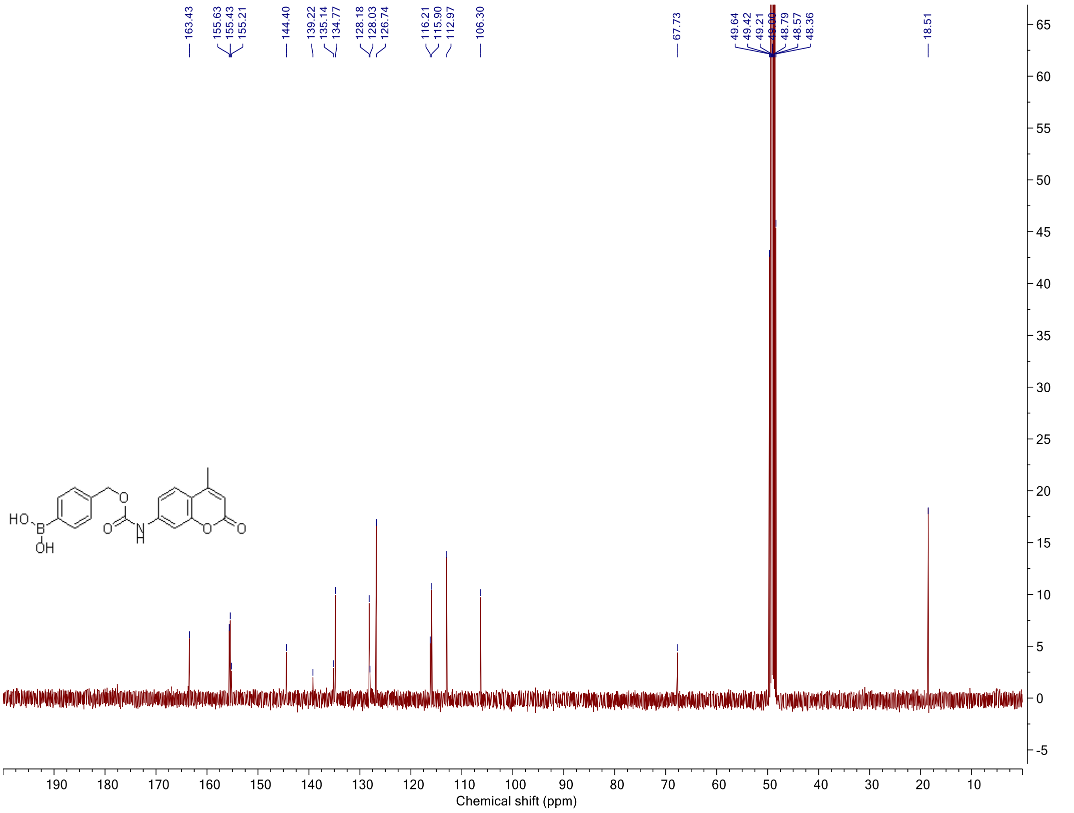


## Synthesis of prodrug BOH-Gem

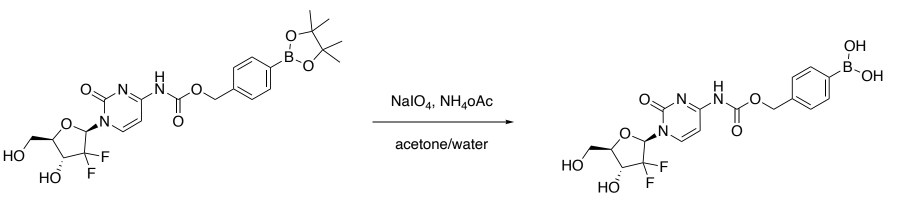


S3 BOH-Gem

Compound S3 was synthesized using the method reported by Matsushita et al.[2] To a solution of compound S3 (100.0 mg, 0.19 mmol, 1 eq.) in 5 mL acetone, $NaIO_4$ (162.5 mg, 0.76 mmol, 4 eq.) and ammonium acetate (58.6 mg, 0.76 mmol, 4 eq.) in 2 mL water were added. The mixture was allowed to stir at room temperature for 16 h. After the completion of the reaction, acetone was evaporated under reduced pressure. The precipitate was collected and washed with deionized water 3 times, to yield a white powder (55.0 mg, 65.2%). $^{1}H$ NMR (400 MHz, MeOD) δ 8.31 (d, $J$ = 7.6 Hz, 1H), 7.76 (d, $J$ = 7.2 Hz, 1NH), 7.63 (d, $J$ = 8.3 Hz, 1H), 7.47 – 7.29 (m, 3H), 6.31 – 6.19 (m, 1H), 5.24 (s, 2H), 4.30 (td, $J$ = 12.2, 8.6 Hz, 1H), 4.02 – 3.91 (m, 2H), 3.87 – 3.74 (m, 1H). $^{13}C$ NMR (101 MHz, MeOD) δ 165.42, 157.42, 154.48, 145.64, 134.96*, 128.23, 126.48, 123.90, 121.32, 97.04, 86.39*, 82.80*, 70.42, 70.19, 69.96, 68.56, 60.26. (* denotes signals affected by $^{13}C$-$^{19}F$ splitting). MS (ESI+) $m/z$ $[M + H]^+$ calculated for $[C_{17}H_{19}BF_2N_3O_8]^+$, 442.12, found 442.01.

HPLC, UV (220 nm) (HPLC Method 2). The peak at 3.1 min is associated to the injection with a cosolvent.

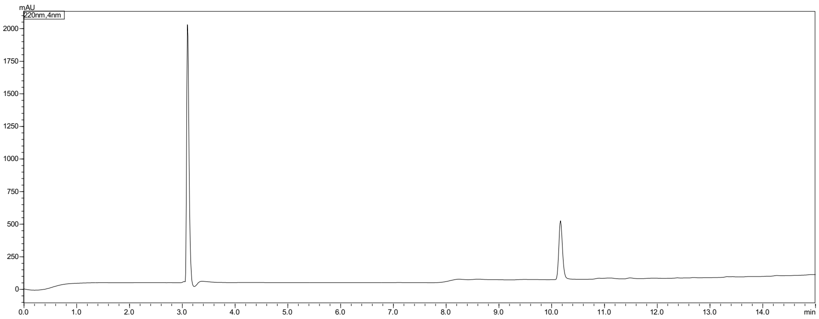


HPLC, UV (275 nm) (HPLC Method 2)

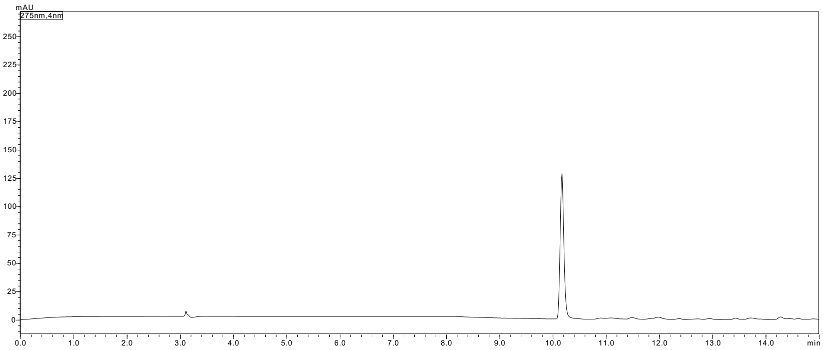

MS TIC (HPLC Method 2)

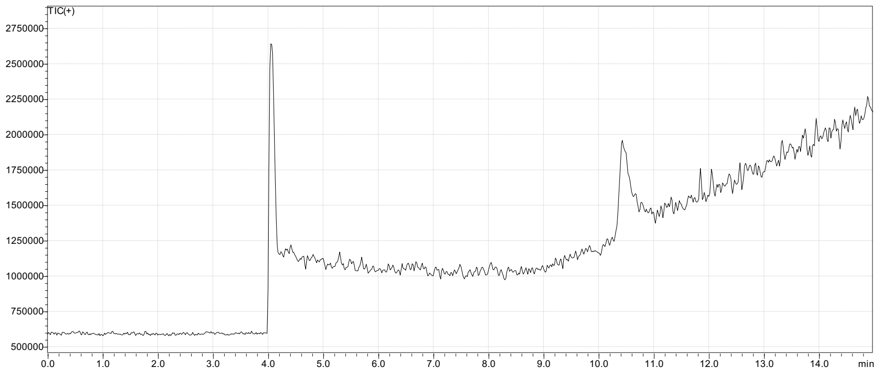


MS (ESI+) *m/z* $[M + H]^+$ calculated for $[C_{17}H_{19}BF_2N_3O_8]^+$, 442.1, found 442.1.

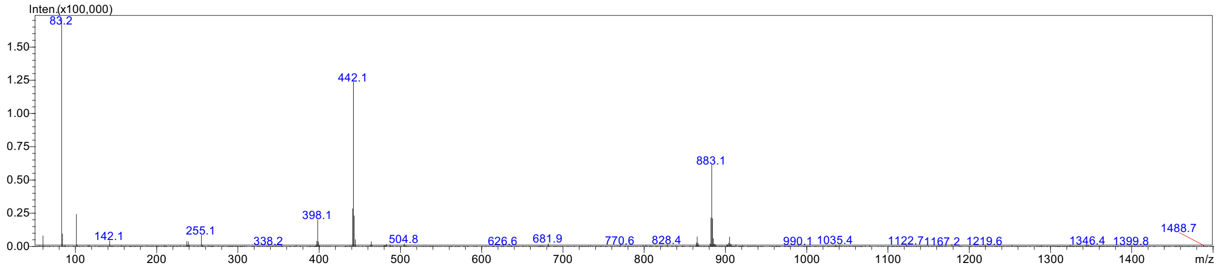


$^{1}$H-NMR (400 MHz, $D_4$-MeOD) spectrum of BOH-Gem

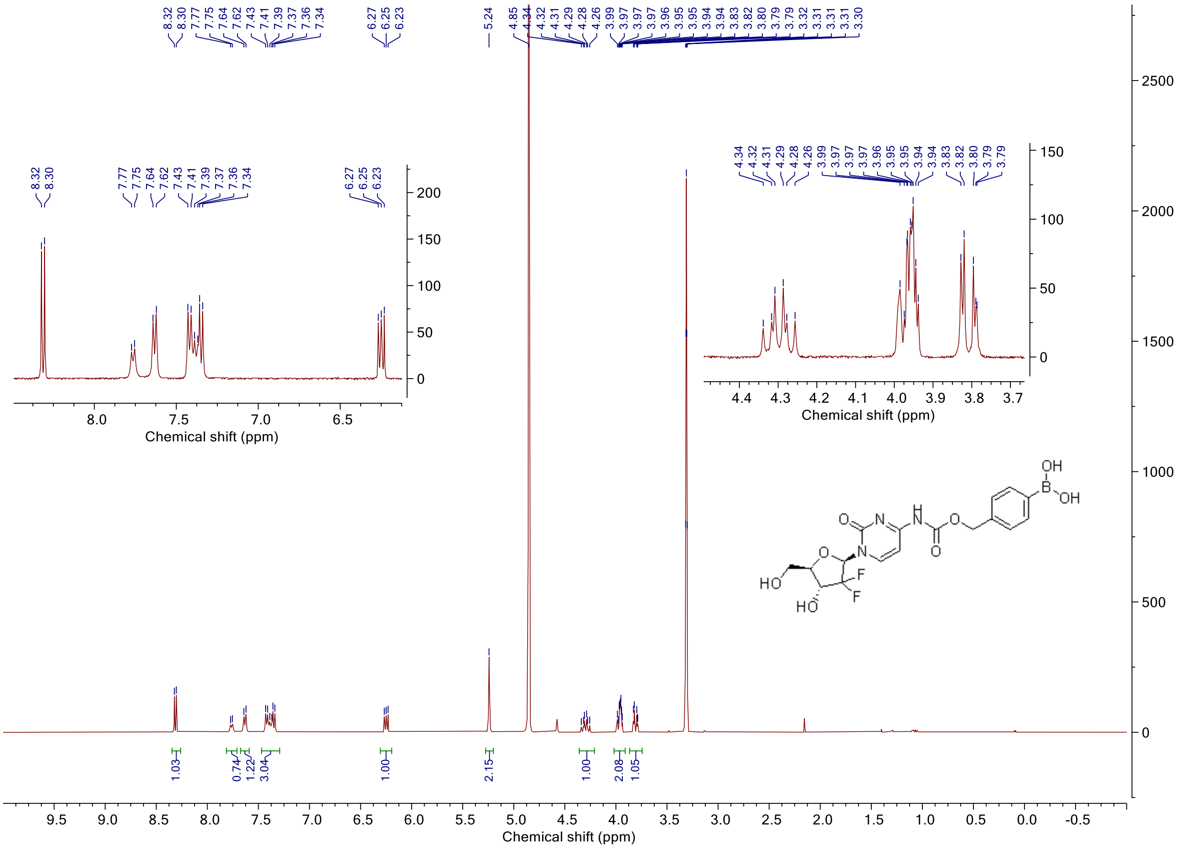

$^{13}$C-NMR (101 MHz, $D_4$-MeOD) spectrum of BOH-Gem

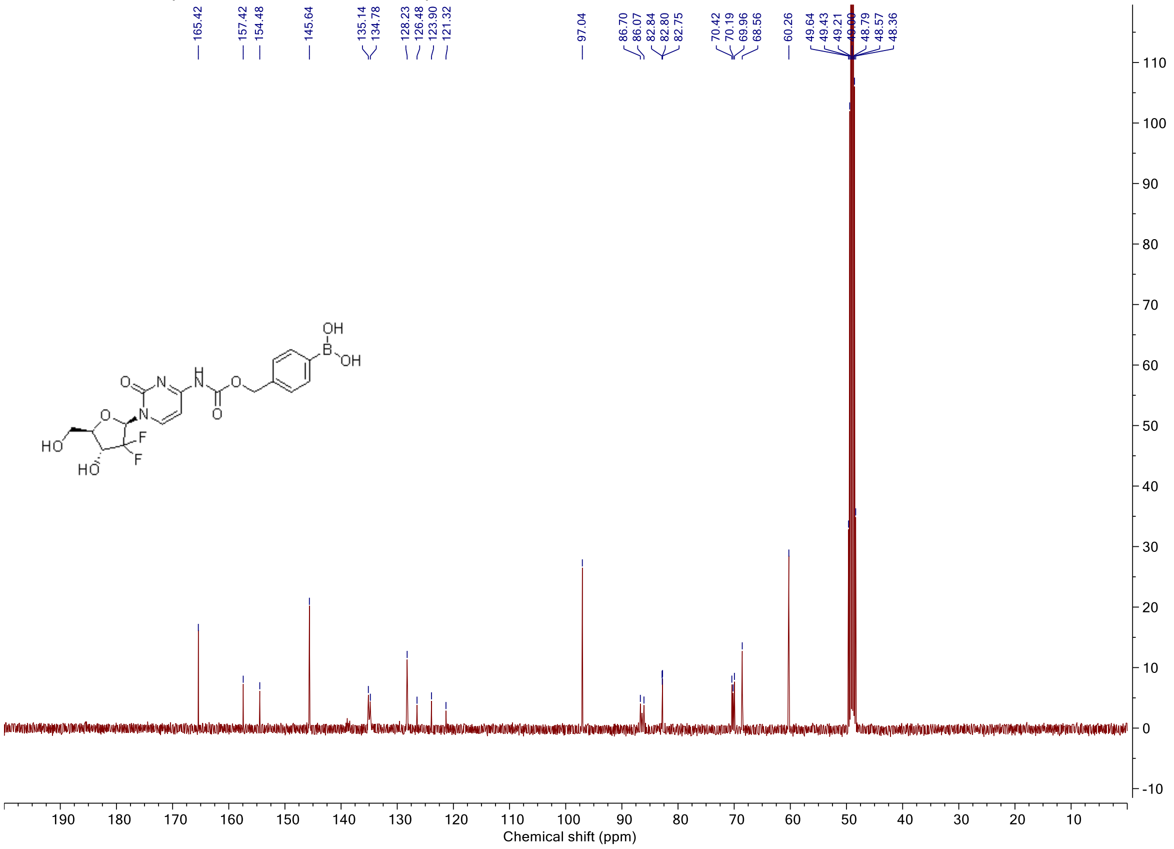


**Preparation and irradiation of BOH-AMC solutions**

A stock solution of BOH-AMC (10 mM) was prepared by dissolving 0.70 mg of BOH-AMC in 0.2 mL of dimethylformamide (DMF). To prepare a 10 μM BOH-AMC solution in phosphate buffered saline (PBS), 1 μL of the stock solution was added to 1 mL of PBS. If required, 1 μL of 2,2,2-trichloroethanol (TCE) was added to 1 mL of the solution to achieve a 12 mM TCE concentration, and 10 μL of a 10 mM amine solution in DMF was added to 1 mL of the PBS solution to reach a final amine concentration of 100 μM. After preparation, solutions were transferred to a Nordion 220 $^{60}$Co gamma cell and irradiated for a specific period to reach a targeted dose.

**Concentration-emission calibration curve of AMC**

A stock solution of AMC (5 mM) was prepared by dissolving 0.28 mg of AMC in 329 μL of DMF. This stock solution was subsequently diluted to the desired concentrations using deionized water. The parameters were set as follows: sensitivity, 600 V; spectrum acquisition starts from 385 nm ends at 500 nm; excited wavelength, 365 nm; response, 4 msec; band width 5 nm; accumulation, 1. The maximum emission intensities at 441 nm were plotted against the concentrations to obtain the concentration-emission calibration curve.

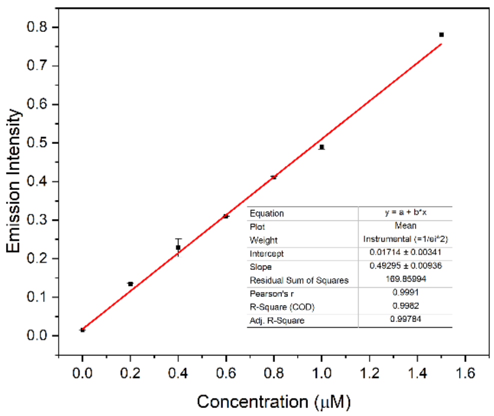


**Figure S1.** The intensity to concentration calibration curve of AMC. Error bars represent experimental variation of three samples.

**Activation of BOH-Gem under different conditions in PBS buffer by X-ray**

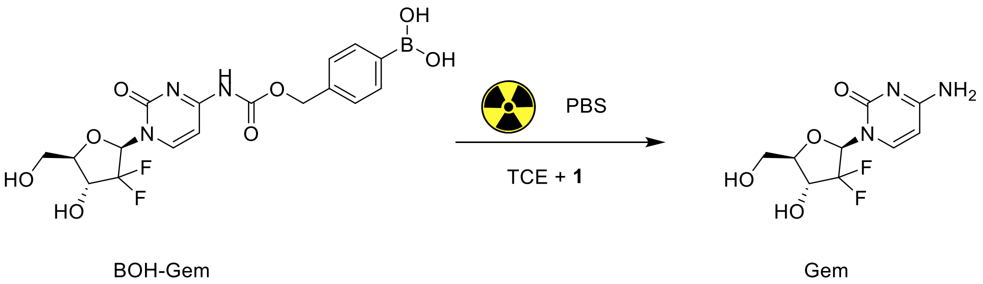


**Preparation and irradiation of BOH-Gem solutions**

Stock solutions of BOH-Gem (10 or 50 mM), Gem (10 or 50 mM) and amine **1** (100 mM) were prepared in anhydrous dimethylformamide (DMF). To prepare a 10 or 50 μM BOH-Gem/Gem solution in phosphate buffered saline (PBS), 1 μL of the stock solution was added to 1 mL of PBS. If required, 1 μL of 2,2,2-trichloroethanol (TCE) was added to 1 mL of the solution to achieve a 12 mM TCE concentration, and 1 μL of a 100 mM amine **1** solution in DMF was added to 1 mL of the PBS solution to reach a final amine concentration of 100 μM.

Solutions were exposed to X-ray irradiation (240 kV, 15 mA) for 6 or 60 Gy and incubated for 30 min after irradiation. Subsequently, the reaction was analysed using HPLC (Method 2, C18, 5 → 95% MeCN in $H_2O$, 0.1% TFA, 15 min, 20 μL injection). Relative quantities of substrate (BOH-Gem) and product (Gem) were determined from the corresponding signals in the UV chromatograms (λ = 275 nm).

**Activation of BOH-Gem determined by HPLC (HPLC Method 2)**

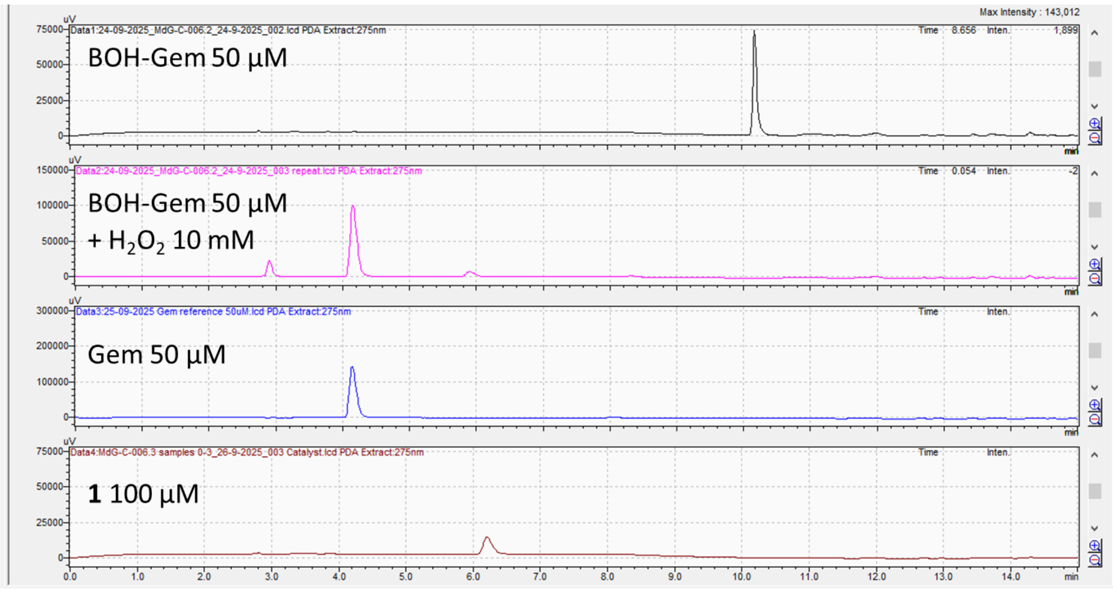


**Figure S2.** HPLC-UV (275 nm channel) chromatographs of (top to bottom) 50 µM BOH-Gem in PBS; 50 µM BOH-Gem + 10 mM $H_2O_2$ in PBS; 50 µM Gem in PBS; 100 µM amine **1** in PBS.

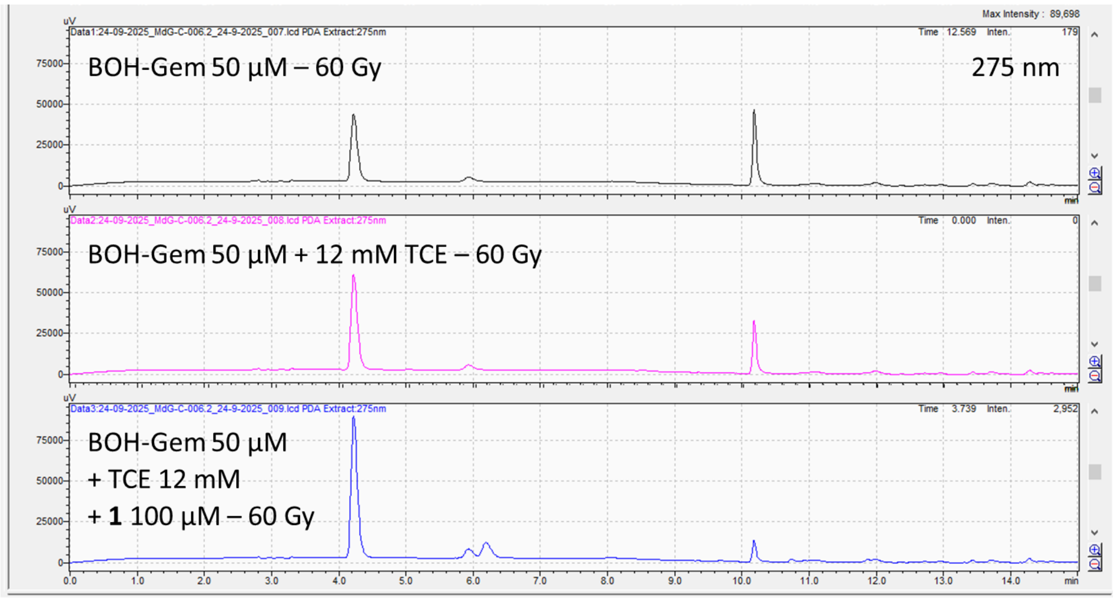


**Figure S3.** HPLC-UV (275 nm channel) chromatographs of 50 µM BOH-Gem after exposure to 60 Gy of X-ray radiation in (top to bottom) PBS; PBS + 12 mM TCE; PBS + 12 mM TCE + 100 µM amine **1**.

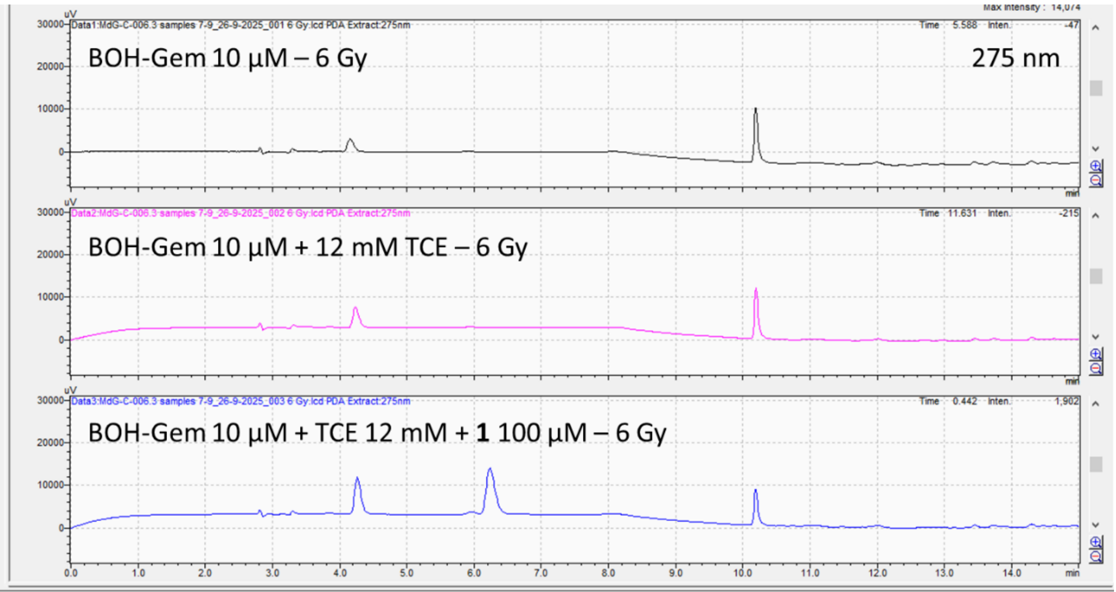


**Figure S4.** HPLC-UV (275 nm channel) chromatographs of 10 µM BOH-Gem after exposure to 6 Gy of X-ray radiation in (top to bottom) PBS; PBS + 12 mM TCE; PBS + 12 mM TCE + 100 µM amine **1**.

**Investigation into the mechanism of the amine-enhanced uncaging reaction.**

**Detection of *N*-oxide after irradiation**

Compound **9** was dissolved in 1 mL water at a concentration of 100 µM. 1 µL of TCE was added to prepare a 12 mM TCE solution. The vials were placed in the centre of the Nordion 220 $^{60}$Co gamma cell. After irradiation, the samples were subjected to HPLC-MS with the mass spectrum detected in positive mode.

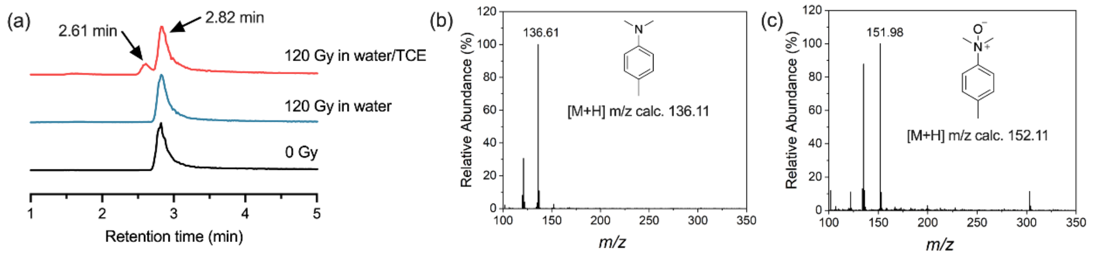


**Figure S5**. (a) HPLC-MS (Method 1) chromatograph (mass channel at selected ions) of amine **9** before exposure to radiation and after irradiation for 120 Gy in water and water/TCE; (b) mass spectrum at 2.82 min; (c) mass spectrum at 2.61 min.

**N-oxide synthesis from compound 9**

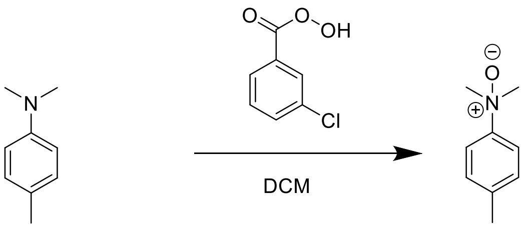


**9**

*N*,*N*-Dimethyl-p-toluidine (9, 54 mg, 0.2 mmol, 1.0 equiv) was dissolved in anhydrous DCM

(2.0 mL) under argon. The solution was cooled to 0°C (ice bath) before adding 3-chloroperbenzoic acid (mCPBA, 138 mg, 75%, 1.5 mmol, 1.5 equiv). The reaction mixture was stirred overnight and allowed to warm to room temperature under argon. The crude reaction mixture was concentrated *in vacuo* and resuspended in anhydrous MeCN (2.0 mL). HPLC-MS analysis (Method 2, C18, 5 → 95% MeCN in $H_2O$, 0.1% TFA, 15 min, 20 µL injection) indicated full conversion from **9** to the corresponding *N*-oxide: MS (ESI+) m/z $[M+H]^+$ calculated for $[C_9H_{14}NO]^+$ 152.1, found 152.2.

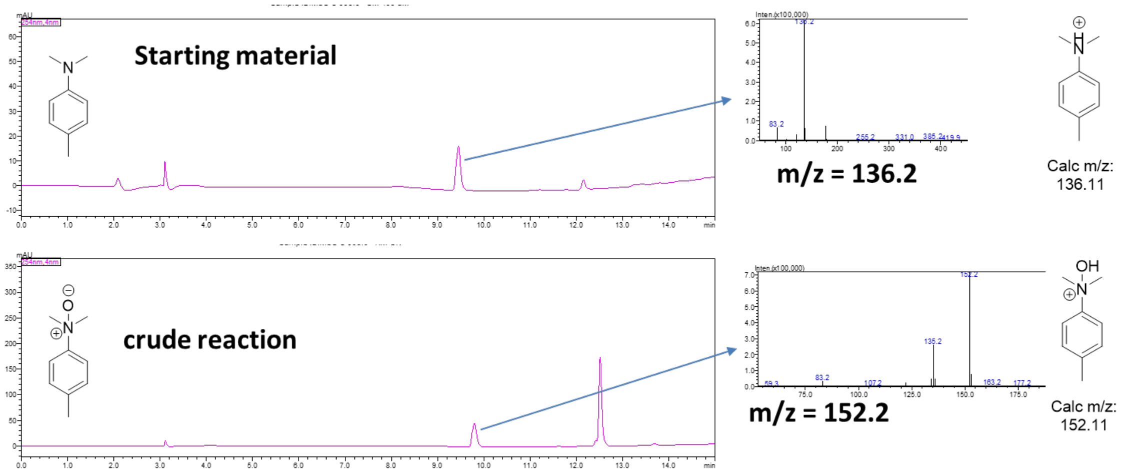


**Figure S6**: LC-MS analysis of the *N*-oxide formation starting from compound **9**. UV chromatograms are extracted at 254 nm. MS (ESI+) analysis is depicted for the selected peaks.

**Uncaging of BOH-AMC with *N*-oxide**

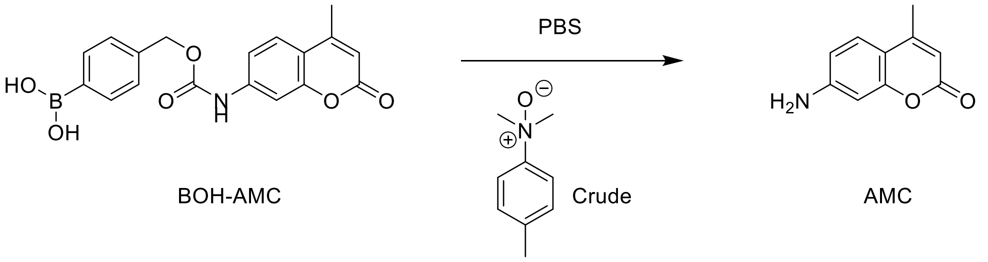


Stock solutions of BOH-AMC (10 mM) and AMC (10 mM) were prepared in anhydrous dimethylformamide (DMF). To prepare a 10 µM BOH-AMC/AMC solution in phosphate buffered saline (PBS), 1 µL of the stock solution was added to 1 mL of PBS. The effect of the crude *N*-oxide of **9** (200 mM in MeCN) was evaluated by adding 0.5 µL to 1 mL of the solution to reach a final *N*-oxide concentration of 100 µM.

Samples were incubated at room temperature for 30 min after adding the *N*-oxide. Subsequently, the reaction was analysed using HPLC (Method 2, C18, 5 → 95% MeCN in $H_2O$, 0.1% TFA, 15 min, 20 µL injection). Relative quantities of substrate (BOH-AMC) and product (AMC) were determined from the corresponding signals in the UV chromatograms (λ = 254 / 330 nm). The reported spectra were obtained with baseline subtraction using spectra obtained with PBS containing 0.1% DMF or 0.1% DMF and 0.05% MeCN.

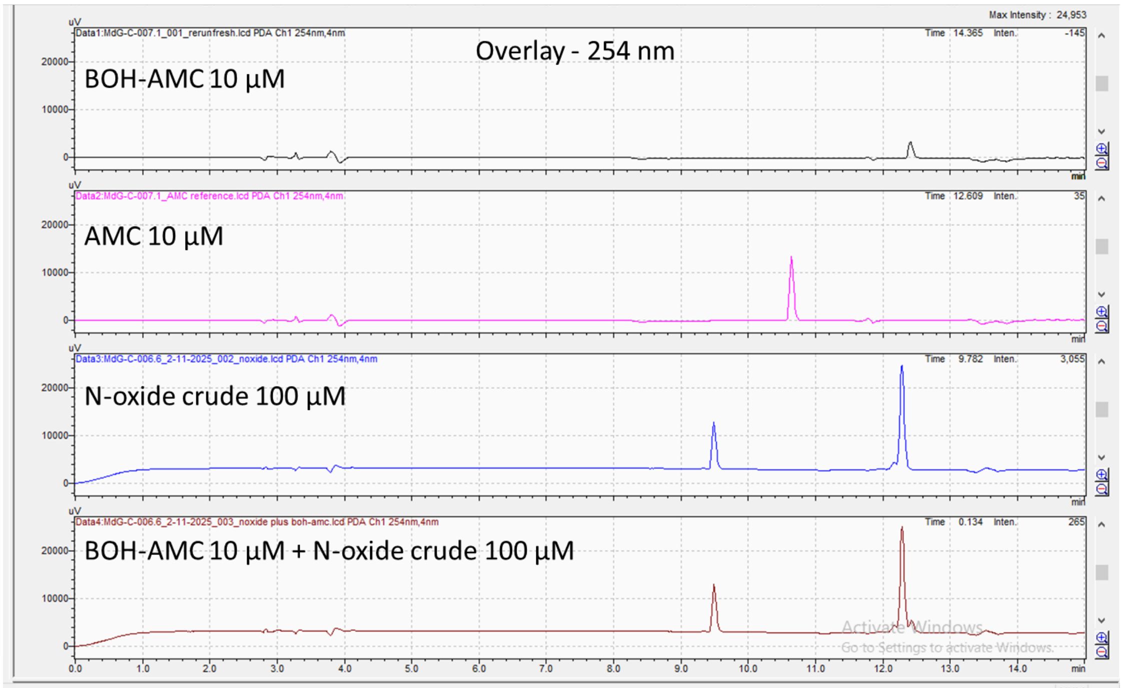


**Figure S7**: HPLC-UV (Method 2, 254 nm channel) analysis of the *N*-oxide driven uncaging of BOH-AMC to AMC. Top to bottom: BOH-AMC; AMC; *N*-Oxide of **9** crude (*N*-oxide at 9.5 min, the peak at 12.3 minutes is likely 3-chloro-benzoic acid); BOH-AMC + *N*-Oxide crude followed by 30 min incubation in PBS.

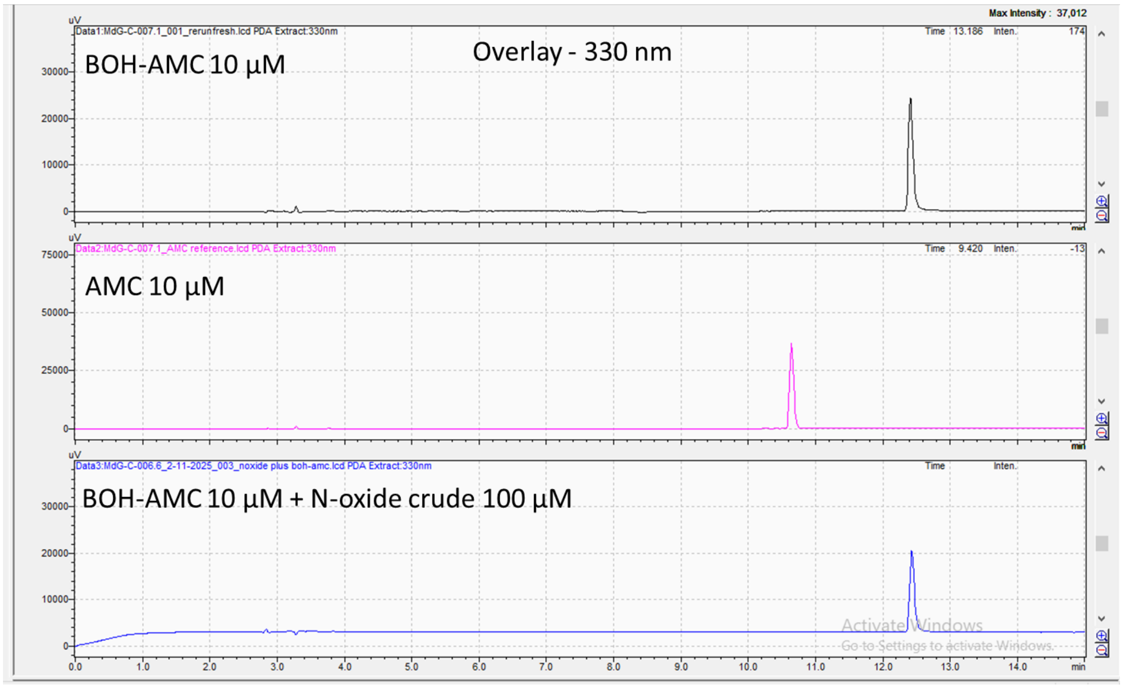


**Figure S8**: HPLC-UV (Method 2, 330 nm channel) analysis of the *N*-oxide driven uncaging of BOH-AMC to AMC. Top to bottom: BOH-AMC; AMC; BOH-AMC + *N*-Oxide crude followed by 30 min incubation in PBS.

## Preparation and irradiation of BOH-AMC solutions

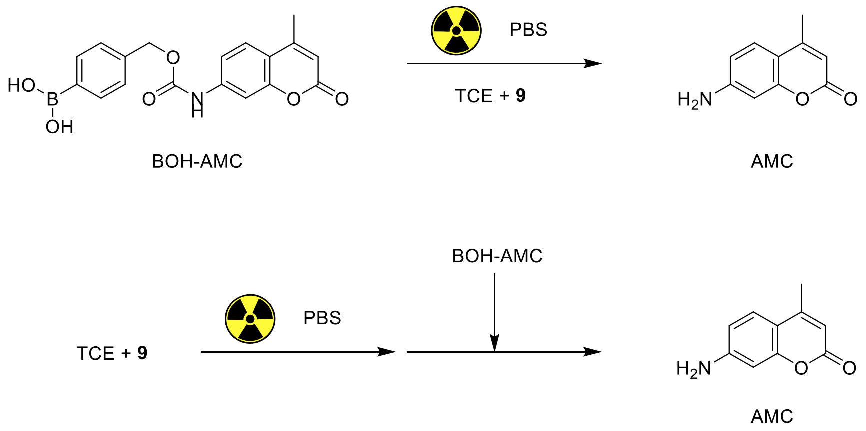


Stock solutions of BOH-AMC (10 mM), AMC (10 mM) and amine **9** (100 mM) were prepared in anhydrous dimethylformamide (DMF). To prepare a 10 µM BOH-AMC or AMC solution in phosphate buffered saline (PBS), 1 µL of the stock solution was added to 1 mL of PBS. If required, 1 µL of 2,2,2-trichloroethanol (TCE) was added to 1 mL of the solution to achieve a 12 mM TCE concentration, and 1 µL of a 100 mM amine **9** solution in DMF was added to 1 mL of the PBS solution to reach a final amine concentration of 100 µM. Solutions were exposed to X-ray radiation (240 kV, 15 mA) for 8 Gy before or after addition of BOH-AMC to differentiate the effects of short-lived reactive intermediates (based on radicals and/or $e^{-}_{aq}$) and stable products of the irradiation ($H_2O_2$ and/or *N*-oxides). Samples were incubated for 30 min at room temperature after irradiation or after adding BOH-AMC. Subsequently, the reaction was analysed using HPLC (Method 2, C18, 5 → 95% MeCN in $H_2O$, 0.1% TFA, 15 min, 20 µL injection). Relative quantities of substrate (BOH-AMC) and product (AMC) were determined from the corresponding signals in the UV chromatograms (λ = 330 nm). The reported spectra were obtained with baseline subtraction using spectra obtained with PBS containing 0.1% or 0.2% DMF.

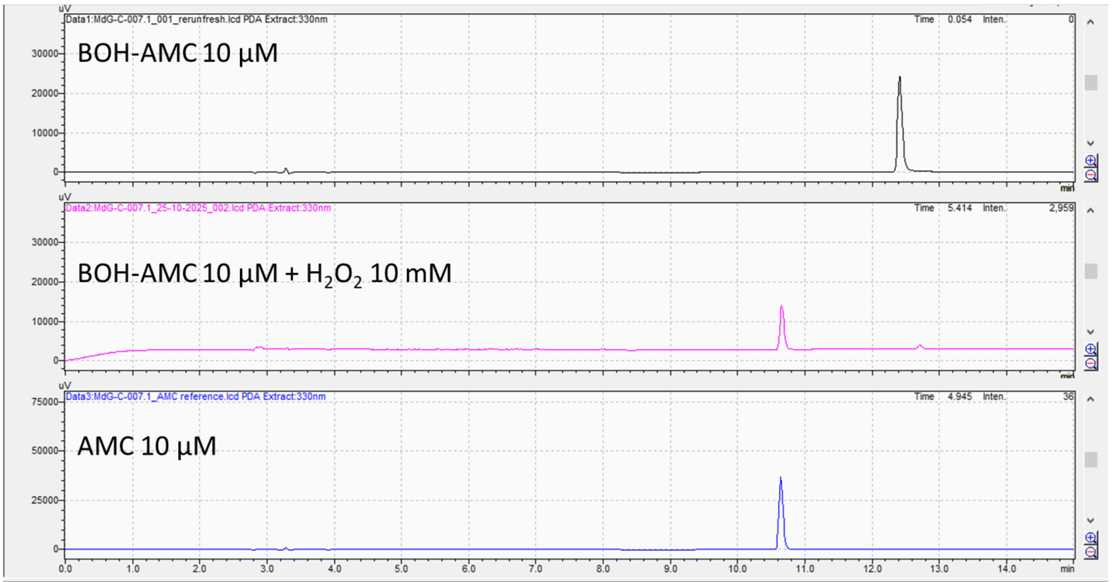


**Figure S9**: HPLC-UV (Method 2, 330 nm channel) of reference compounds. Top to bottom: BOH-AMC; BOH-AMC + 10 mM $H_2O_2$ (to ensure uncaging); AMC.

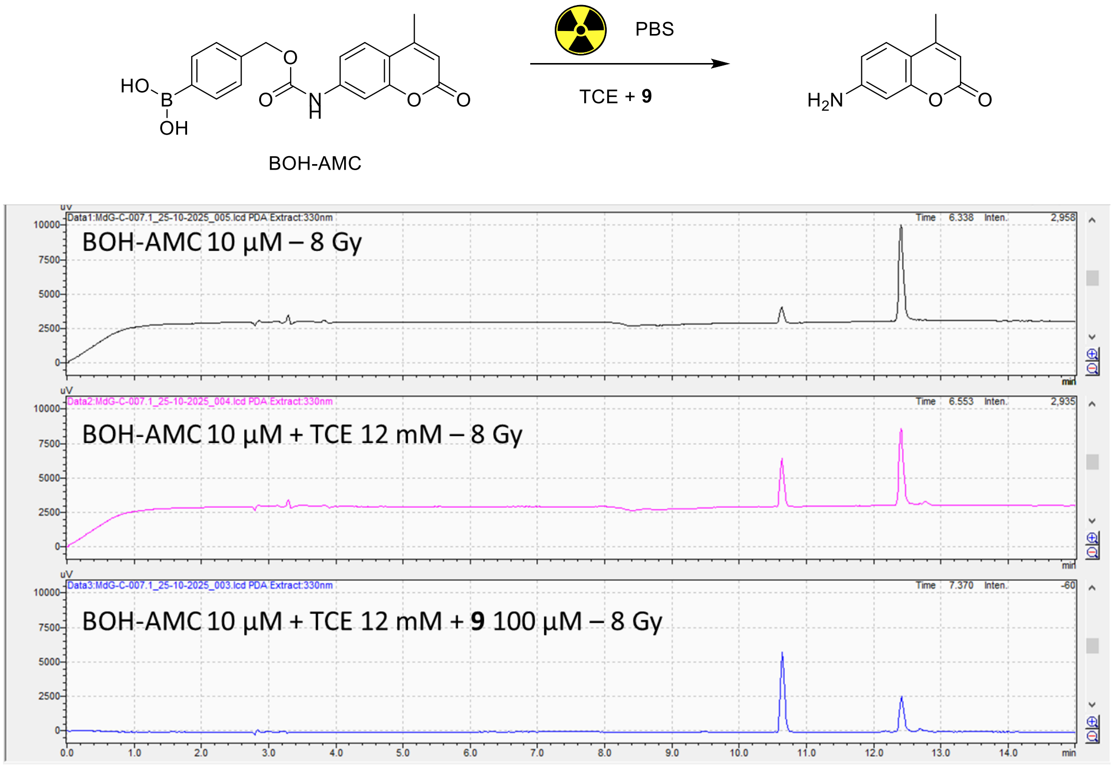


**Figure S10**: HPLC-UV (Method 2, 330 nm channel) of irradiated samples (8Gy X-ray) in the **presence** of BOH-AMC. Top to bottom: BOH-AMC; BOH-AMC + TCE; BOH-AMC + TCE + amine **9**.

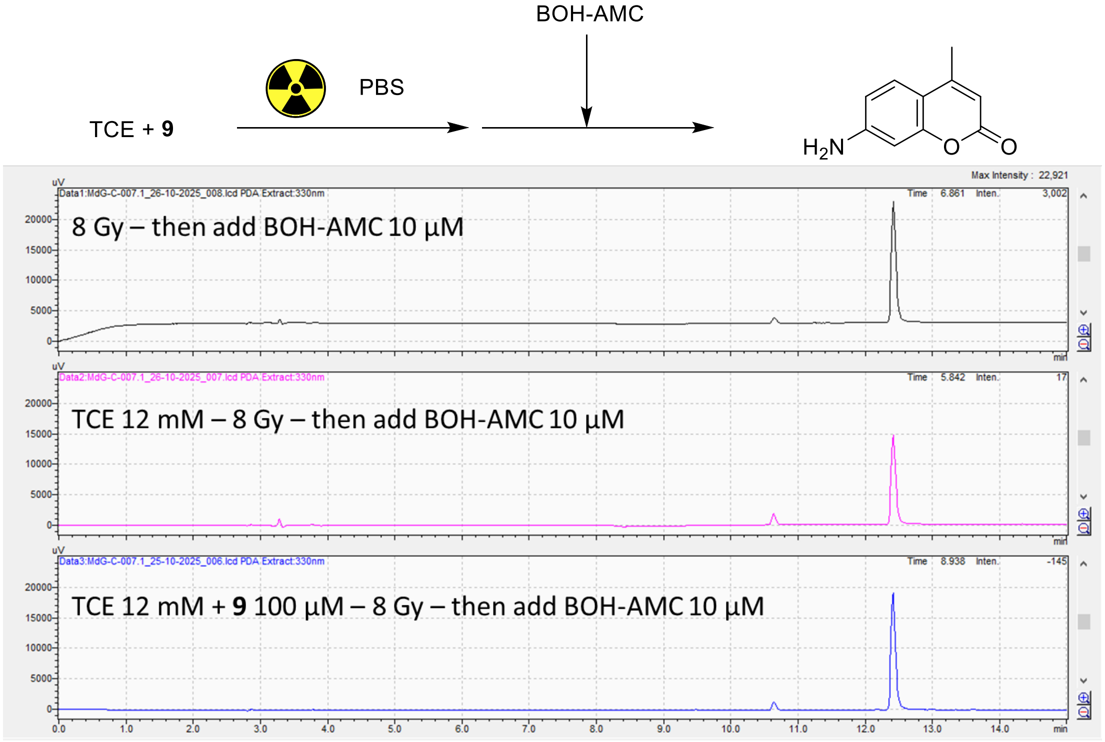


**Figure S11**: HPLC-UV (Method 2, 330 nm channel) of irradiated samples (8Gy X-ray) in the **absence** of BOH-AMC. Top to bottom: PBS; PBS with TCE; PBS with TCE and amine **9**. BOH-AMC is added 30 min after irradiation.

**Oxygen determination**

To vary the concentration of oxygen in water, we bubbled a mixture of nitrogen and oxygen to the solution for 10 min. The partial pressure of oxygen was adjusted by regulating the flow rates of oxygen and nitrogen via a mass-flow-controller. Specifically, the nitrogen flow rate was maintained at 15 mL/min, while the flow rate of oxygen was adjusted accordingly to achieve the desired ratio. The exact concentrations of oxygen were determined using a Clark electrode, following the method reported by Pouvreau et al.[3] PBS was stirred under atmospheric conditions for 10 minutes to ensure saturation with air. The air saturated PBS was added to the cell with Clark electrode, and the polarization voltage was recorded. Then, 10 mg sodium hydrosulfite ($Na_2S_2O_3$) was added to the cell and the polarization voltage at 0% oxygen was recorded. The polarization voltages of air-saturated PBS and sodium hydrosulfite contained PBS were calibrated as 21% and 0% oxygen, respectively. The polarization voltages of samples were measured, and the voltages were calibrated to molar concentrations. The concentration of oxygen in air-saturated (21%) PBS at 25 °C (256 μM) was used to convert from molar concentration to partial pressure. The calculated oxygen partial pressures and the concentrations are detailed in **Table S1**.

**Table S1**. Calibration of oxygen concentration using Clark electrode.

| Partial pressure in gas line (%) | Voltage (V) | Partial pressure (%) | concentration (μM) |
|---|---|---|---|
| 5 | 0.122 ± 0.004 | 4.45 ± 0.06 | 54.2 ± 1.2 |
| 2 | 0.022 ± 0.001 | 2.24 ± 0.01 | 27.4 ± 0.2 |
| 0 | -0.059 ± 0.008 | 0.43 ± 0.10 | 5.3 ± 2.0 |
| 21* | 0.868 | 21 | 256* |
| $NaS_2O_3$ | -0.079 | 0 | 0 |

* Values from Zhang et al.[4]

**Preparation of BOH-AMC solutions with varying oxygen concentrations**

BOH-AMC solution with targeted concentrations of amine and TCE were prepared as described before. The solution was transferred into a 1.5 mL vials with septum cap. The samples were bubbled with controlled flow of oxygen and nitrogen through needles. For the samples with 21% oxygen, no bubbling process was applied. After regulating the partial pressure, the needles were removed and the septum cap was sealed with grease.

**IC50 viability assay**

U87 cells (human glioblastoma cell line) were cultured in Dulbecco's modified Eagle's medium (DMEM, Gibco) containing 10% fetal bovine serum (Biowest) and 1% penicillin/streptomycin (VWR) in a cell incubator (Heracell®, Heraeus) with a humidified atmosphere containing 5% $CO_2$ at 37 °C.

8000 U87 cells were seeded in 96-well plates overnight. Gemcitabine and BOH-Gem were dissolved in DMSO to obtain stock solutions of 10 mM, respectively. Afterwards, the stock solution of Gem and Pro-G were diluted to 100, 10, 1, 0.1, 0.05, 0.025, 0.01, 0.001 μM with DMEM. Subsequently, 100 μL of DMEM containing various concentrations of drug were added into cells. After 48 hours incubation, cells were washed with PBS twice, followed by

adding 100 µL Cell Counting Kit-8 (CCK-8, Dojindo Laboratories, Japan)/DMEM = 1:9. The viability of the cells was determined by the absorbance at 450 nm wavelength by a microplate scanning spectrophotometer (PowerWave XS™, Bio-Tek). Each concentration was tested in six replicates.

**Fluorescent probe activation upon irradiation**

U87 cells were seeded in 8-well chamber slides at a density of $1\times10^4$ cells per well. A 10 mM stock solution of BOH-AMC was prepared in DMSO. Cells were incubated overnight with a treatment mixture containing 12 µM BOH-AMC, 500 µM TCE, and 5 µM amine **1**. After incubation, cells were exposed to X-ray radiation (240 kV, 15 mA) for 20 Gy, and returned to incubator for a 3-hour recovery period. The cells were then washed three times with PBS and fixed with 4% paraformaldehyde. As a control, cells were treated with 4 µM coumarin (equivalent to the yield of AMC released after 20 Gy of radiation), incubated overnight and washed three times with PBS and fixed with 4% paraformaldehyde.

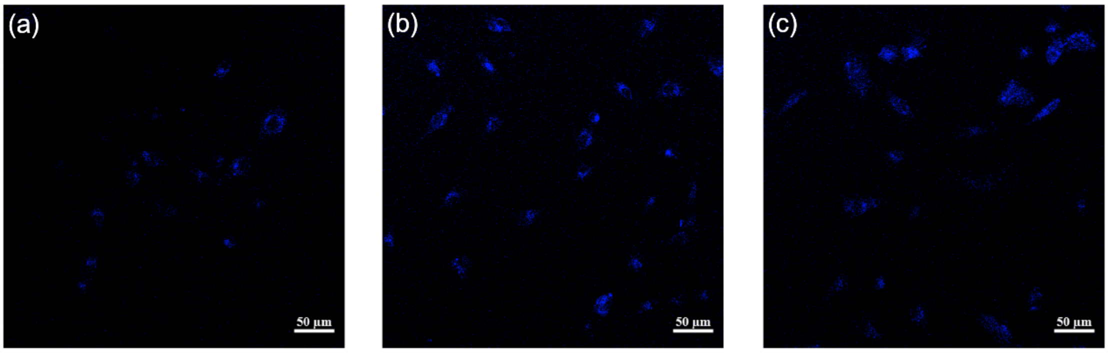


**Figure S12**. Confocal laser scanning microscope images of U87 cells (a) treated with 12 µM BOH-AMC, light intensity per surface area is 407.2 $mm^{-2}$ processed by ImageJ; (b) treated with 12 µM BOH-AMC, 500 µM TCE, 5 µM amine **1** and 20 Gy of X-ray irradiation, light intensity per surface area is 600.4 $mm^{-2}$ and (c) treated with 4 µM AMC, light intensity per surface area is 566.0 $mm^{-2}$. Scale bar 50 µm.

**2D cell viability assay**

Gemcitabine and BOH-Gem were dissolved in DMSO to get stock solutions of 10 mM. The stock solutions were diluted to 100 µM using PBS, and subsequently diluted to 20 nM using DMEM or DMEM containing 500 µM TCE and 5 µM amine **1**.

8000 U87 cells were seeded in 96 well plates and incubated overnight. The solutions added to the cells were: control, 100 µL of DMEM; A+T, 100 µL DMEM containing 500 µM TCE and 5 µM amine **1**; BOH-Gem, 100 µL DMEM containing 20 nM BOH-Gem; BOH-Gem+A+T, 100 µL DMEM containing 20 nM BOH-Gem, 500 µM TCE and 5 µM amine **1**; Gem+A+T, 100 µL DMEM containing 20 nM Gem, 500 µM TCE and 5 µM amine **1**. After treatments, cells were exposed to X-ray irradiation (240 kV, 15 mA) for 6 Gy. After 48 hours incubation, cells were washed with PBS twice, followed by adding 100 µL CCK-8/DMEM = 1:9 to test the viability. The viability of non-irradiated groups was normalized to “control”, and the viability of irradiated group was normalized to “6 Gy” group. Each group was tested in six replicates.

**Spheroid experiments**

1. Cytotoxicity of BOH-Gem under X-ray radiation compared to Gem.

2000 U87 cells were seeded in the pre-coated U shape 96 well plates (Greiner BioOne, CELLSTAR®) and allowed to grow for 3-4 days. 100 μM Gem or BOH-Gem were prepared as mentioned above and were diluted to desired concentrations by DMEM containing both 500 μM TCE and 5 μM amine **1**. Once the spheroids reached a volume of 300-400 $\mu m^3$, various concentrations of Gem or BOH-Gem were added to the cells. The spheroids were then irradiated by the X-rays for 6 Gy. After 48 h of treatment, the drug was removed by washing the cells twice with PBS, followed by the addition of fresh DMEM. The cell media were exchanged on the day 6. The growth of the spheroids was assessed at different timepoints using a binocular microscope. ImageJ software was used to measure the spheroid size.

2. The enhanced drug release by additional TCE and amine **1**

To assess the promoting effect of TCE and amine **1** on the release efficiency of BOH-Gem, several experimental groups were set. These included: Gem + A + T (Gem in DMEM with 500 μM TCE and 5 μM amine **1**), BOH-Gem + A + T (BOH-Gem in DMEM with 500 μM TCE and 5 μM amine **1**), BOH-Gem + T (BOH-Gem in DMEM with 500 μM TCE), BOH-Gem (BOH-Gem in DMEM) and control (DMEM with 500 μM TCE and 5 μM amine **1**). 2000 U87 cells were seeded in the pre-coated U shape 96 well plates (Greiner BioOne, CELLSTAR®) and allowed to grow for 3-4 days. 100 μM Gem or BOH-Gem were prepared as mentioned above and were diluted to desired concentrations by DMEM containing both 500 μM TCE and 5 μM amine **1**. Once the spheroids reached a volume of 300-400 $\mu m^3$, various concentrations of Gem or BOH-Gem were added to the cells. The spheroids were then irradiated by the X-rays for 6 Gy. After 48 hours, the drug was removed by washing the cells twice with PBS, followed by the addition of fresh DMEM. The cell culture media were refreshed on the day 6 and day 12. The growth of the spheroids was assessed at different timepoints using a binocular microscope. ImageJ software was used to measure the spheroid diameter.

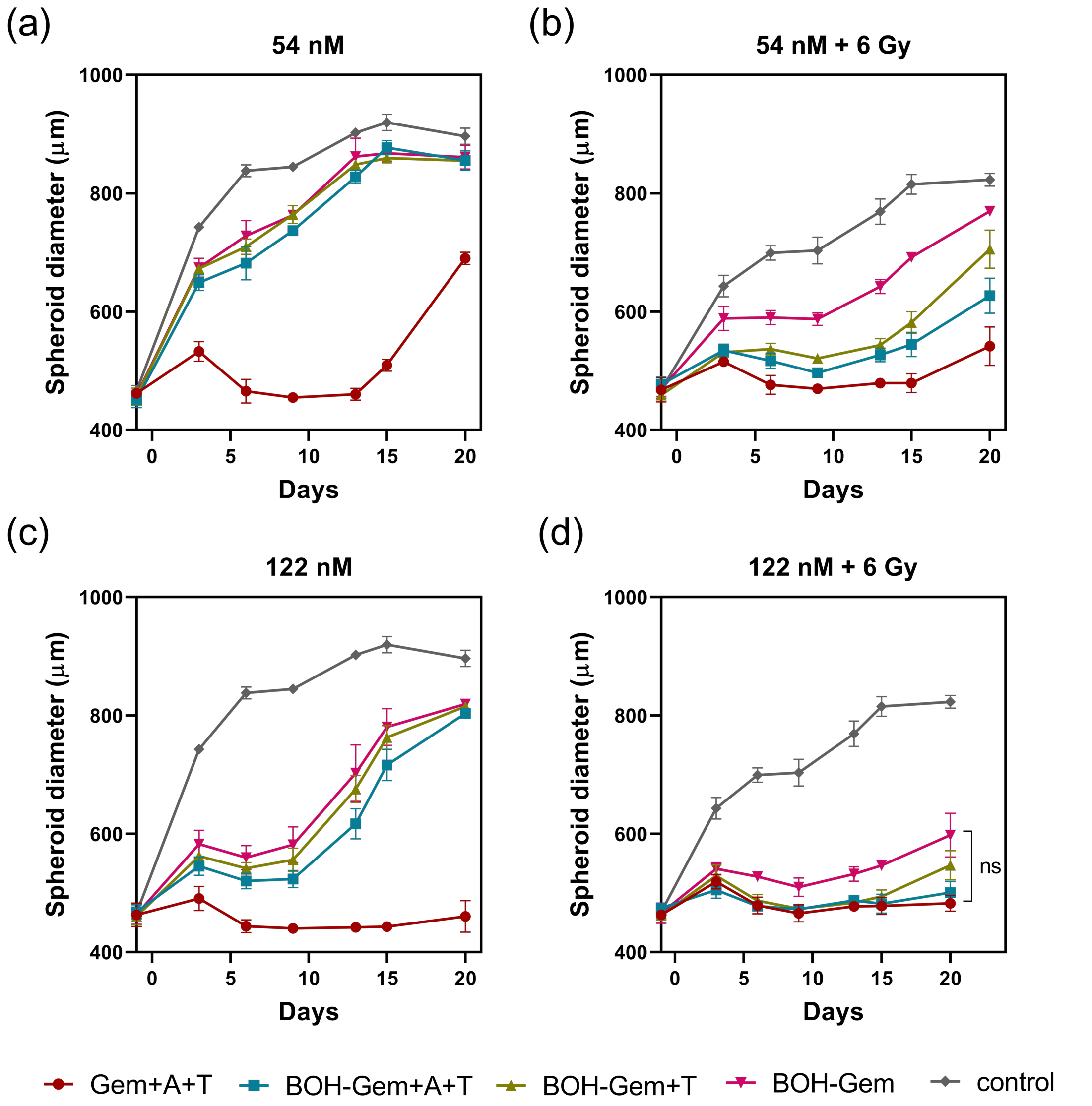


**Figure S13**. The diameter of spheroids after various treatments as a function of time. (Gem+A+T: addition of gemcitabine, 5 μM amine **1** and 500 μM TCE; BOH-Gem+A+T: addition of prodrug, 5 μM amine **1** and 500 μM TCE; BOH-Gem+T: addition of prodrug and 500 μM TCE; BOH-Gem: addition of prodrug; Control: addition of PBS). (a) the concentrations of BOH-Gem and Gem were 54 nM; (b) the concentrations of BOH-Gem and Gem were 54 nM, cell spheroids were exposed to 6 Gy of X-rays; (c) the concentrations of BOH-Gem and Gem were 122 nM; (d) the concentrations of BOH-Gem and Gem were 122 nM, cell spheroids were exposed to 6 Gy of X-rays.

## Reference

(1) Yao, T.; Luthjens, L. H.; Gasparini, A.; Warman, J. M. A study of four radiochromic films currently used for (2D) radiation dosimetry. *Radiation Physics and Chemistry* **2017**, *133*, 37-44.

(2) Matsushita, K.; Okuda, T.; Mori, S.; Konno, M.; Eguchi, H.; Asai, A.; Koseki, J.; Iwagami, Y.; Yamada, D.; Akita, H.; et al. A Hydrogen Peroxide Activatable Gemcitabine Prodrug for the Selective Treatment of Pancreatic Ductal Adenocarcinoma. *ChemMedChem* **2019**, *14*, 1384-1391.

(3) Pouvreau, L. A.; Strampraad, M. J.; Van Berloo, S.; Kattenberg, J. H.; de Vries, S. NO, $N_2O$, and

$O_2$ reaction kinetics: scope and limitations of the Clark electrode. *Methods Enzymol* **2008**, *436*, 97-112.
(4) Xing, W.; Yin, M.; Lv, Q.; Hu, Y.; Liu, C.; Zhang, J. Oxygen Solubility, Diffusion Coefficient, and Solution Viscosity. In *Rotating Electrode Methods and Oxygen Reduction Electrocatalysts*, 2014; pp 1-31.
